# α‑Aminoboronic Acid Moieties in Boro Dipeptides Modulate Proteasome Subunit Selectivity and Provide Access to Compounds with Potent Anticancer and Anti-Inflammatory Activity

**DOI:** 10.1021/acs.jmedchem.5c02548

**Published:** 2025-12-04

**Authors:** Nika Strašek Benedik, Andrej Šterman, Lara Smrdel, Stane Pajk, Stanislav Gobec, Zdenko Časar, Martina Gobec, Izidor Sosič

**Affiliations:** a Faculty of Pharmacy, 37663 University of Ljubljana, Aškerčeva Cesta 7, SI-1000 Ljubljana, Slovenia; b Lek Pharmaceuticals d.d., Sandoz Development Center Slovenia, Verovškova Ulica 57, SI-1526 Ljubljana, Slovenia

## Abstract

Proteasomes regulate cellular protein homeostasis and are key targets in treating cancer, inflammation, and autoimmune diseases. The two main forms, the constitutive proteasome and immunoproteasome, each contain three catalytically active subunits with distinct substrate specificities. The first approved proteasome inhibitor, bortezomib, is nonselective and causes dose-limiting toxicity. Herein, we report dipeptide boronic acids with varying P1 residues, prepared using our recently developed method for α-aminoboronic acids formation. Most compounds inhibited various immuno/proteasome subunits in the low nanomolar range, displaying inhibition profiles distinct from bortezomib, ranging from β5i/β1i-selective to β5c/β5i-directed inhibitors. Although their cytotoxicity to cancer cells was not improved compared to bortezomib, selected compounds proved less toxic to noncancer cells and with anti-inflammatory activity comparable to that of zetomipzomib (KZR-616). The presented boro dipeptides with tailored P1 residues provide a basis for designing subunit-selective compounds with boronic acid as the warhead and optimized P2 and/or P3 positions.

## Graphical abstract

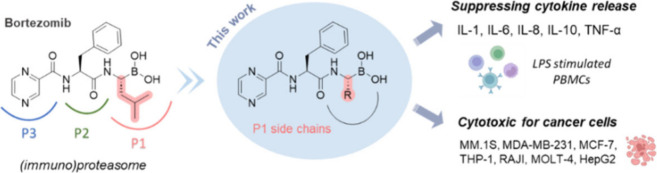

## Introduction

As a part of the ubiquitin-proteasome system, the 26S proteasome is crucial for the maintenance of cellular protein homeostasis by rapidly degrading misfolded or damaged proteins, and slow degradation of most other intracellular proteins.^1–5^ It regulates many cellular processes, including immune response, signal transduction, genome integrity, and apoptosis.^6–9^ The 26S proteasome consists of regulatory 19S subunits and proteolytic 20S core complex (the core particle, CP), which encompasses three active subunits with different catalytic activity and substrate specificity: β1c, β2c, and β5c.^10–14^ In humans, there are two major types of 20S core: the constitutive proteasome (cCP), which is found in all cell types, and the immunoproteasome (iCP), which is normally found in cells of hematopoietic origin and in most other tissues after the stimulation by interferon-γ and tumor necrosis factor-α (TNF-α).^15–17^ In the iCP, the standard catalytically active subunits are replaced by their inducible counterparts, i.e., β1i, β2i, and β5i. This leads to changes in cleavage specificity as iCP has a higher affinity for the cleavage of peptides with hydrophobic and basic residues.^18^ Because cancer cells are metabolically highly active, they rely heavily on proteasome-mediated protein degradation and enter apoptotic processes when proteasome function is impaired.^19,20^ In addition, proteasomes have a key role in the activation of the nuclear factor kappa-light-chain-enhancer of activated B cells (NF-κB), which is important in inflammatory response and pathogenesis of cancer.^21,22^ These facts provide a scientific basis for proteasome inhibition in the treatment of neoplastic, inflammatory, and autoimmune diseases.^23–25^ Through selective inhibition of either cCP or iCP (or both), which have, as noted above, three different catalytic subunits each, the type of pharmacological activity and side effect profiles of novel inhibitors might be regulated more precisely.^26,27^

Several proteasome inhibitors are being used for the treatment of multiple myeloma, and others are currently in different stages of clinical studies.^28^ Bortezomib was the first marketed proteasome inhibitor (FDA approval in 2003),^29^ followed by carfilzomib,^30^ and ixazomib.^31^ These compounds covalently inhibit the proteasome, but differ in the nature of the interaction with Thr1 at the *N*-terminal active site: while the epoxyketone warhead of carfilzomib irreversibly inhibits the proteasome,^32^ the boronic acid in bortezomib and ixazomib forms a reversible covalent bond with the nucleophilic Thr within the catalytically active cCP and iCP subunits.^33^ The use of both reversible and irreversible covalent inhibitors is an attractive modality in medicinal chemistry,^34–36^ and this notion served as a basis for our previous work, in which we investigated several different warheads in immuno/proteasome inhibitors.^37–40^ Since the α-aminoboronic acid in bortezomib and ixazomib represents a well-validated moiety for proteasome inhibition, further investigation of this interesting compound class serves as an attractive drug development approach. The development of new α-aminoboronic acid–based analogs might also mitigate some of the drawbacks of bortezomib, such as off-target effects^41,42^ and peripheral neuropathy.^43,44^ Bortezomib and ixazomib consist of three building blocks, which can be labeled as P1, P2, and P3, where P1 stands for l-boro-Leu, P2 for l-Phe or Gly, respectively, and P3 for pyrazine-2-carboxylic acid or 2,5-dichlorobenzoic acid (Figure 1A).

**Figure 1:**
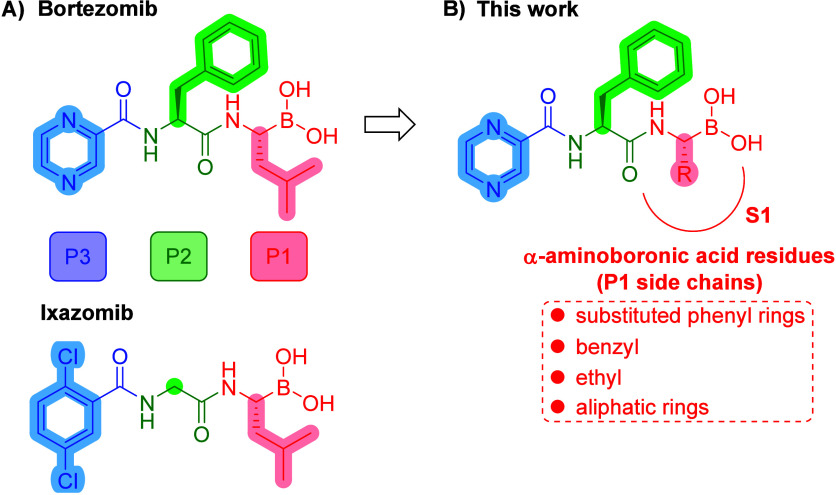
(A) Chemical structures of dipeptide boronic acids bortezomib and ixazomib with P1, P2, and P3 side chains (P sites) highlighted. One can observe that ixazomib was developed simply by optimization of P2 and P3 residues of bortezomib. (B) A general structure of derivatives described in this work.

Of note, the structure–activity relationships (SARs) of bortezomib derivatives have been well researched, but focused almost exclusively on modifications at the P2 and P3 residues,^45–50^ since the required building blocks are readily available. On the other hand, while the synthesis of α-aminoboronic acids and their derivatives was first developed in the 1980s,^51–53^ the access to these valuable compounds remains challenging.^54–56^ This is probably the reason why relatively few boro dipeptides with variations at the P1 position have been synthesized and evaluated for their proteasome inhibition.^57,58^ However, crystallographic analyses suggest that by modifying the side chain of the α-aminoboronic-acid P1 residue, different cCP and iCP subunit inhibition profiles could be achieved due to subtle differences in the binding channels of active sites.^59–61^ We therefore synthesized a limited series of bortezomib analogs with distinct P1 side chains (Figure 1B) and determined their IC_50_ values for the human cCP and iCP subunits. The compounds showed variable potencies and subunit binding preferences, which resulted in different effects on the viability of several cancer cell lines, as well as on the ability to reduce cytokine production in lipopolysaccharide (LPS)-stimulated peripheral blood mononuclear cells (PBMCs).

## Results and Discussion

Recently, we developed a novel procedure for the synthesis of α-aminoboronic acids from potassium acyltrifuoroborates (KATs)^62–64^ by catalytic hydrogenation of benzyl trifluoroborate iminiums (TIMs).^65^ We then advanced this methodology by asymmetric hydrogenation of primary TIMs **2** to primary trifluoroborate ammoniums **3**, followed by their conversion to chiral *N*-deprotected α-aminoboronic acids **4** using hexamethyldisiloxane (HMDSO) in MeOH with aqueous HCl (Scheme 1).^66^ With a series of structurally diverse enantioenriched α-aminoboronic acids in hand, we were able to readily prepare novel boro dipeptides described herein using an established^67^ solid-phase peptide synthesis protocol (Scheme 1).

**Scheme 1:**
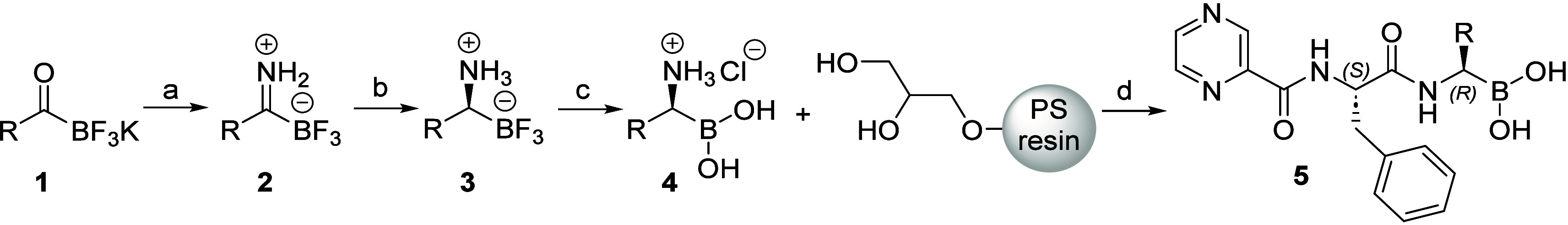
Synthetic Methodology toward Bortezomib Analogs^a^

a The procedures to obtain α-aminoboronic acids **4** are described in detail in our recent report^66^ and available in the Experimental Section. The final scope of compounds was limited to the availability of KATs at the onset of our study. Reagents and conditions: (a) NH_4_Cl (5 equiv), MeOH, 40 °C, 1–6 h; (b) for **3a**–**3h**: [(*R*,*R*)-TethTsDpen-RuCl] (2 mol %), 20 bar H_2_, MeOH, rt, 20 h; for **3i**–**3l**: [(*S*,*S*)-TethTsDpen-RuCl] (4 mol %), 20 bar H_2_, MeOH, rt, 72 h; (c) HMDSO (3 equiv), 2 M HCl (*aq*, 4 equiv), MeOH, 2–4 h; (d) (1) DMF, rt, 16 h; (2) Fmoc-l-Phe-OH (3 equiv), HATU (3 equiv), IPEA (5 equiv), DMF, rt, 2 h; (3) 10% piperidine/DMF, 10 + 5 min; (4) pyrazine-2-carboxylic acid (3 equiv), HATU (3 equiv), DIPEA (5 equiv), DMF, rt, 2 h; (5) CH_2_Cl_2_:MeOH:H_2_O = 5:4:1, 3 × 30 min. Note: Because the enantiomeric ratios (*R*/*S*) between both enantiomers of **3** ranged from 92:8 to 99:1,^66^ this resulted in the final compounds **5** being present in two diastereomeric forms, whereby the desired (*R*,*S*)-**5** diastereomer was prevalent and (*S*,*S*)-**5** diastereomer was minor. The diastereomeric ratios ranged from 82:18 to 99:1. For this calculation, we used the diastereotopic CH-Bn proton, which was the most discernible between both forms.

We synthesized 12 bortezomib analogs with distinct side chains at the P1 position, which varied in polarity, steric bulk, and electronic density (compounds **5a**–**5l**, Figure 2), thus enabling different interactions in the S1 pockets of active sites of cCP and iCP subunits. With the aim to obtain as much SAR data as possible, the inhibitory activities of compounds were evaluated against all catalytically active subunits of the human cCP and iCP by determining their IC_50_ values using subunit-specific fluorogenic substrates (Table 1).

**Figure 2:**
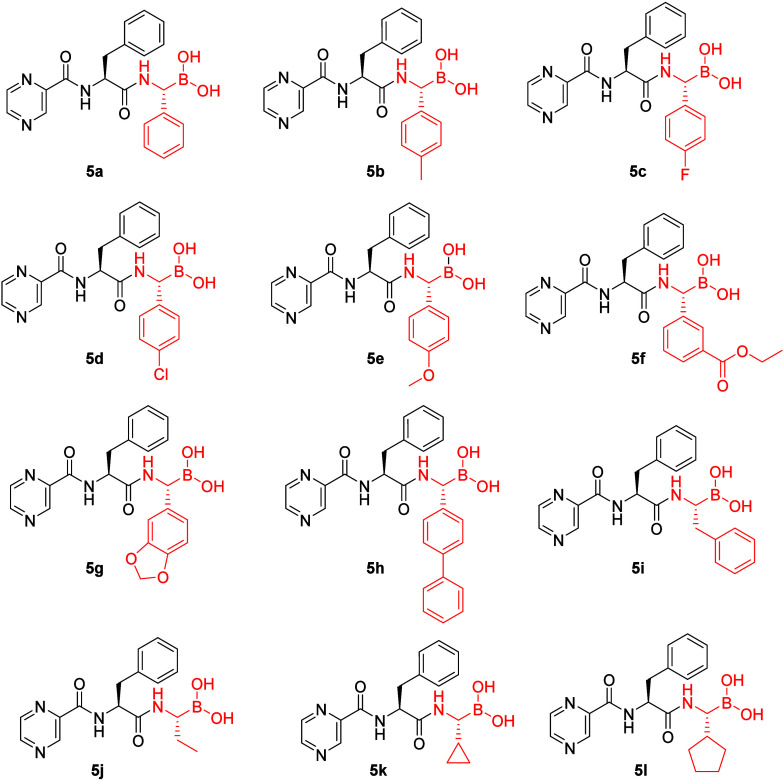
Structures of P1-derivatized boro dipeptides. Among these, **5a** (P1-phenyl), **5b** (P1-*p*-tolyl), **5d** (P1-*p*-chlorophenyl), **5i** (P1-benzyl), and **5j** (P1-ethyl) were described previously, but their inhibitory activities were not comprehensively assessed across all six subunits of both cCP and iCP.^57,58^ Of note, the focus of studies with previously disclosed analogs was *Mycobacterium tuberculosis* proteasome (**5a**, **5b**, **5d**, and **5j**) and mycobacterial caseinolytic proteases P1 and P2 (**5i**).

**Table 1:**
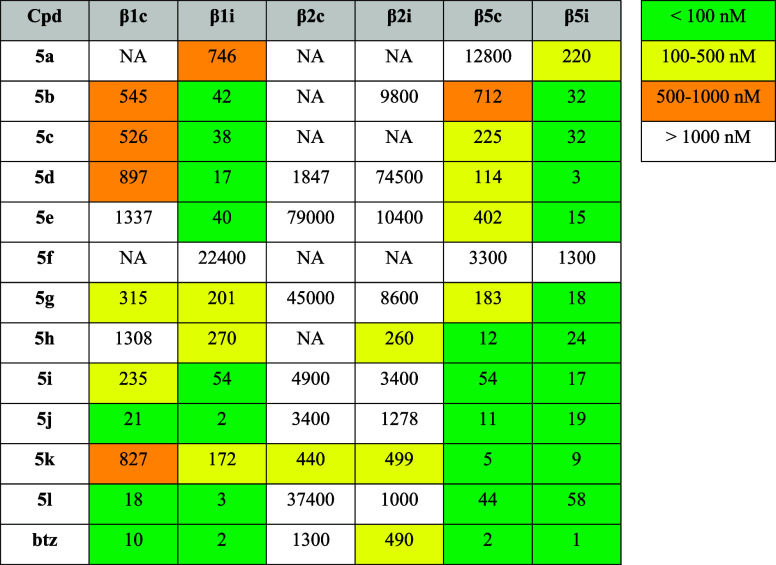
Inhibitory Potencies of Compounds against All Subunits of the Human cCP and iCP^a^

a Activities are reported as mean IC_50_ values (nM) from at least three independent experiments. The IC_50_ values with SDs are available in the Supporting Information (Table S1). For comparison, the IC_50_ values for bortezomib (btz) were determined and these were in accordance with literature data.^68^ We also determined inhibitory activities of other control compounds used in this study (Table S2). NA: not active at 50 μM concentration.

Since all compounds were modified only at the α-aminoboronic acid residue, we were able to thoroughly assess the contributions of these moieties to the inhibitory properties. As expected, variations of the side chain at the P1 position had marked effects on both potency and inhibition preferences across different subunits. Because of limited number of compounds, and to avoid overinterpretation of the SAR data, only apparent inhibition trends are summarized here. Similar to bortezomib, the inhibitory activities of the tested compounds toward β2 subunits, apart from **5h** and **5k**, were negligible. Compounds **5a**–**5e** with an aromatic α-aminoboronic acid residue showed preference for the inhibition of β1i and β5i subunits, especially when bearing an additional substituent at the *para* position. Derivatives **5b** (P1-*p*-tolyl), **5c** (P1-*p*-fluorophenyl), and **5e** (P1-*p*-methoxyphenyl) showed inhibition of β1i and β5i at low nanomolar concentrations, while inhibiting the cCP counterparts with 10–20-fold lower potencies. Compound **5d** (P1-*p*-chlorophenyl) had a similar inhibitory profile, but with an even better IC_50_ for the β1i (17 nM, 50-fold lower than for β1c) and β5i subunits (3 nM, 30-fold lower than for β5c). Of note, while **5a** (P1-phenyl) was generally less potent, it still inhibited subunits β1i (746 nM) and β5i (220 nM) the most. Interestingly, compound **5f** was a poor inhibitor altogether, most probably due to the bulky ester group at the *meta* position of the phenyl ring (Table 1).

Analog **5g** with a 3,4-methylene­dioxyphenyl side chain at the P1 position showed preference for β5i (IC_50_ = 18 nM, 10-fold lower than for β1i, β1c, and β5c, Table 1), while **5h** with a bulky P1-biphenyl preferentially inhibited both β5 subunits with IC_50_ values of 12 nM (β5c) and 24 nM (β5i), which was 10–20-fold better than its IC_50_ for subunit β1i (270 nM) and even more in comparison to the IC_50_ value for β1c, which exceeded 1 μM (Table 1). Interestingly, this is the only compound that inhibited subunit β2i better than bortezomib (IC_50_ value of 260 nM vs 490 nM), with no detectable inhibition of its cCP counterpart. Adding a degree of flexibility via a methylene moiety (compound **5i**, P1-benzyl) allowed this substituent to conform into the S1 pockets of β5c, β5i, and β1i subunits, inhibiting them to a similar extent with IC_50_s ranging from 17–54 nM.

Derivatives **5j** (P1-ethyl), **5k** (P1-cyclopropyl), and **5l** (P1-cyclopentyl) are structurally most similar to bortezomib. The inhibitory profiles of **5j** and **5l** were similar to that of bortezomib as they favored the β1 and β5 subunits of both proteasomes, whereby a marginal decrease in potency was observed (Table 1). It should be noted that this could be due to the presence of approximately 10% of the inactive (*S*,*S*)-diastereomers in the final compounds. Namely, it has already been established that inverting the boron stereocenter has a detrimental effect on proteasome inhibition.^69^ A different subunit preference profile, however, was achieved with **5k**, which was comparable to bortezomib in its inhibition of β5c and β5i (IC_50_ values of 5 nM and 9 nM, respectively), while it inhibited β1c and β1i with approximately 80-fold lower potency. Compound **5k** was also the only derivative that inhibited both β2 subunits with IC_50_ values of 440 nM (β2c) and 499 nM (β2i) (Table 1).

When the results are examined from the perspective of the previously described structural data and the established cleavage patterns of β1c/i, β2c/i, and β5c/i subunits, the trend of more potent β1i inhibition (compared to β1c) by our compounds seems reasonable as it has been demonstrated that the β1i substrate binding channel is preferentially targeted by compounds bearing hydrophobic residues at the P1 position.^59,60^ The fact that compound **5l** (P1-cyclopentyl) showed very potent β1c inhibition is somewhat surprising, because S1 pocket of β1c generally disfavors large hydrophobic residues.^70^ We found it a bit unfortunate that no potent β2c/i inhibitors were discovered, given that β2 subunits should also accommodate nonpolar P1 residues.^12^ We postulate that notable inhibition of β2c and β2i can only be achieved by concurrent and high-affinity binding into S2 and/or S3 pockets. The enlarged S1 pocket of the β5i subunit and its preference for aromatic residues^60,71^ was evident also from our data, as analogs **5a**–**5g** with aromatic P1 residues proved to be better β5i inhibitors compared to β5c. Similar β5c and β5i inhibitory potencies of P1-biphenyl- (**5h**) and P1-benzyl-bearing compounds (**5i**), therefore, seem counterintuitive and structural studies are needed to fully explain this phenomenon. On the other hand, although the S1 pocket of β5c is smaller, the fact that it has been shown to efficiently bind P1-Leu decorated inhibitors^59,60^ it was not surprising that it was also able to accommodate similarly sized P1-cyclopropyl and P1-cyclopentyl moieties of **5k** and **5l**, respectively.

We next investigated the effects of the selected boro dipeptides **5b**, **5c**, **5j**–**l** on the viability of several cancer cell lines (Figure 3) and compared them to β1i-selective (KZR-504, ML604440), β2i-selective (Lu-002i), β5i-selective (M3258, DPLG3), β1i/β5i-targeting (KZR-616), and β5c/β5i-targeting (ONX-0914) inhibitors, as well as to pan-proteasome inhibitors bortezomib and carfilzomib. The structures of the control compounds are shown in Figure S11.

**Figure 3:**
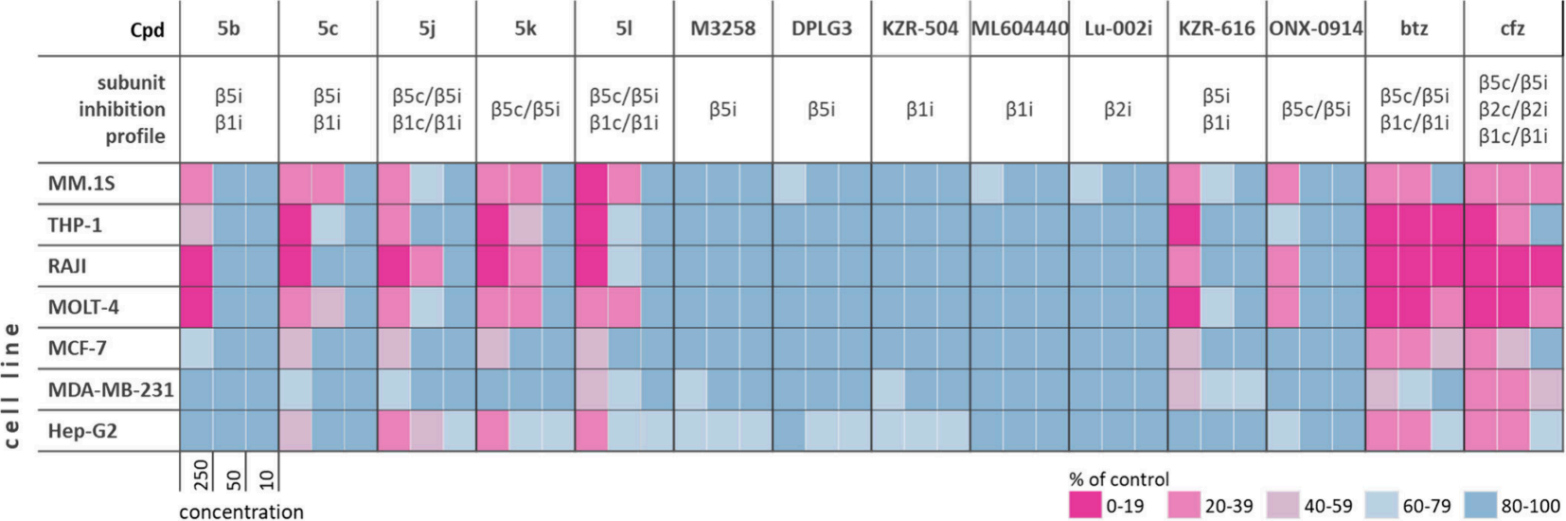
Heatmap depicting cytotoxic effects of selected boro dipeptides and a number of subunit selective control compounds. The compounds and control cCP and iCP inhibitors were tested at 10, 50, and 250 nM. The cells were treated with compounds at indicated concentrations for 72 h. Cytotoxicity was determined by MTS assay and was normalized to DMSO controls. The bar graph representation of data with means ± SD is shown in the Supporting Information (Figures S3–S9). In the heatmap, mean of three independent experiments (*N* = 3) is depicted. MM.1S, multiple myeloma cell line; THP-1, acute monocytic leukemia cell line; RAJI, Burkitt’s lymphoma cell line; MOLT-4, acute lymphoblastic leukemia cell line; MCF-7, breast cancer cell line; MDA-MB-231, triple negative breast cancer cell line; Hep-G2, hepatocellular carcinoma cell line. Note 1: **5d** and **5h** were not used in cell-based assays due to limited solubility. Note 2: the subunit inhibition profiles for individual compounds are designated if IC_50_ is below 100 nM.

Compounds were evaluated at three concentrations (10, 50, and 250 nM) with the aim to have a better estimation regarding the contributions of particular subunit(s) inhibition on the cell-killing effect (i.e., the goal was to span compound concentration range so that those were in the range of IC_50_ values for one or more subunits). The immediate conclusion that can be deduced from Figure 3 is that control compounds that are selective inhibitors of individual iCP subunits had negligible effect on cytotoxicity against all cancer cells, even at 250 nM, a concentration which significantly exceeds their IC_50_ values for the corresponding subunit. The effect of M3258 (87% viable cells at 250 nM, Figure S3), which was developed for the treatment of multiple myeloma,^68^ in MM.1S was consistent with previous findings, where the IC_50_ value of 367 nM for the reduction of MM.1S viability was reported.^72^ Interestingly, the β1i/β5i-selective KZR-616 (zetomipzomib),^73^ which is undergoing clinical trials for the treatment of lupus,^74^ showed notable cytotoxicity, albeit only at 250 nM and with a preference for hematological cell lines. Only slightly reduced, yet similar effects were observed for β5c/β5i-targeting ONX-0914 at 250 nM. On the other hand, pan-proteasome inhibitors were very potent at 50 nM in all cell lines (and in some cases at 10 nM) (Figure 3 and Figures S3–S9), with carfilzomib being superior to bortezomib, which is consistent with previous observations.^20,75^ As expected, bortezomib analogs **5b** and **5c** (β1i/β5i-selective) showed a similar profile to KZR-616 at the highest concentration tested, whereas **5c** was slightly more potent at 50 nM in MM.1S and MOLT-4 cells (Figure 3, Figures S3 and S6). Given the broader cCP and iCP inhibition by **5j**–**5l**, it was also not surprising that these compounds exhibited more potent cytotoxicity in all cell lines. It should be noted that the analog **5k** (P1-cyclopropyl) demonstrated equipotent effects (or even more potent in THP-1 cells) compared to **5j** and **5l**, although it is a poorer β1c/i inhibitor. Although speculative without experimental evidence, one of the possible reasons for this could be that **5k** at 250 nM also marginally inhibited both β2 subunits, thereby achieving better cytotoxicity, as described previously.^20,76–78^ Nevertheless, the cell-killing effects of the new P1-modified boro dipeptides did not supersede those of bortezomib and carfilzomib. Based on the cCP and iCP inhibition results of control compounds (Table S2) and novel boro dipeptides (Table 1), we believe that significantly more potent inhibition of the β2 sites is imperative to further improve cancer cell toxicity and increase efficacy in solid tumors.^79^

Numerous studies have reported the efficacy of bortezomib in different models of inflammation and immune-mediated disorders.^80–83^ Despite its enormous potential for the treatment of these diseases, all reviews cited above conclude that the key factor for future usefulness is to minimize bortezomib-related toxicity, especially if a potential (new) drug treatment would be chronic.^25^ To evaluate the anti-inflammatory potential of our set of boro dipeptides, we determined whether the compounds reduce cytokine secretion in LPS-stimulated human PBMCs (Figure 4). Prior to that, we demonstrated that compounds **5** showed no significant cytotoxic effect on PBMCs and human umbilical vein endothelial cells at all concentrations tested, whereas bortezomib was found to be more cytotoxic (Figures S1 and S2). Derivatives **5b**, **5c**, **5j**–**l** and a set of controls were then assayed at 250 nM, where no nonspecific cytotoxicity was observed and where full inhibition of individual or combined cCP and/or iCP subunits was expected for compounds with IC_50_ values below 100 nM. Of note, the effects observed and described below are most probably due to inhibitory effects of compounds solely on the iCP subunits, because PBMCs essentially express only the iCP with negligible cCP levels present (Figure S10).

**Figure 4:**
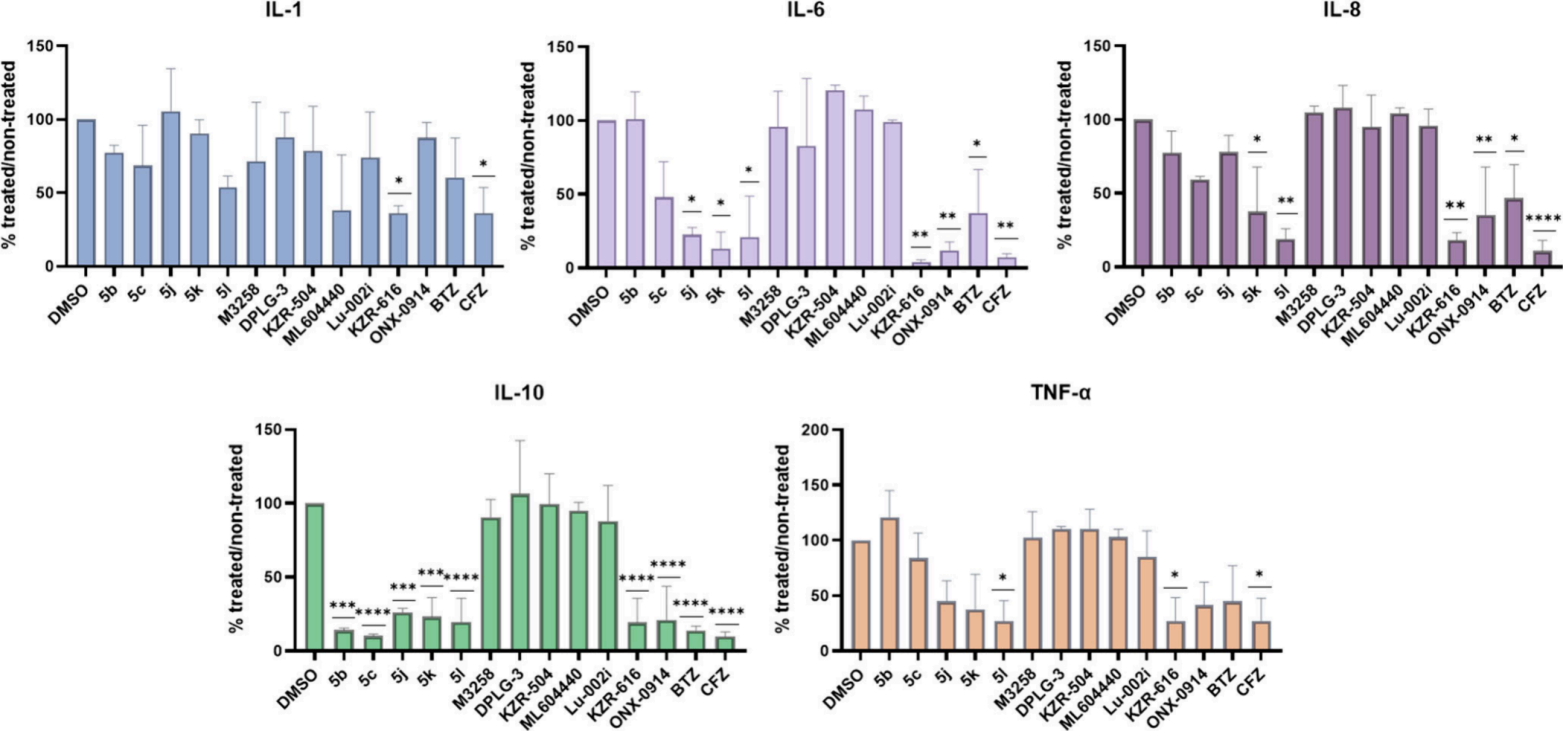
Effect of compounds **5b**, **5c**, and **5j**–**l**, as well as subunit selective control compounds on the secretion of cytokines in LPS-stimulated PBMCs. All compounds were tested at 250 nM. The cells were pretreated for 1 h with inhibitors, followed by the addition of LPS (1 μg/mL). As the negative control (designated by ‘DMSO’ in figure legends), cells were treated only with DMSO, followed by the addition of LPS (1 μg/mL). The concentrations of cytokines were determined in the supernatants after additional 24 h treatment. The results are represented as means ± SD of four independent experiments (*N* = 4). Statistical significance between untreated controls versus treated was calculated using one-way ANOVA post hoc Dunnett’s test. A *p*-value of less than 0.05 was considered significant (****p < 0.0001; ***p < 0.001; **p < 0.01; *p < 0.05).

In perfect accordance with previous findings,^73,79,84,85^ we demonstrated that β1i-, β2i-, and β5i-subunit selective inhibitors fail to inhibit cytokine secretion in LPS-stimulated PBMCs (Figure 4). As expected, the dual β1i/β5i inhibitor KZR-616 reduced the secretion of IL-1, IL-6, IL-8, IL-10, and TNF-α to a significant degree at 250 nM (Figure 4). Very similar result was obtained for ONX-0914, despite the fact that this compound is deemed as β5c/β5i-selective. Given its irreversible nature of binding to the proteasome and still notable inhibition of β1i subunit (IC_50_ = 336 nM, Table S2) *in vitro*, we postulate that under the conditions tested this subunit was substantially coinhibited in PBMCs. Interestingly, analogs **5b** and **5c** showed significantly diminished ability to affect cytokine release (with the exception of equipotent effect on IL-10) in comparison to KZR-616, despite having the same subunit inhibition preferences (Figure 4). On the other hand, compounds **5j**–**l**, which also potently inhibit subunits β1i and β5i, significantly reduced the production of cytokines IL-6, IL-8, IL-10, and TNF-α. Bortezomib showed similar or slightly weaker effects compared to the synthesized analogs, whereby some of these effects could also be due to minor toxicity in PBMCs (Figure S1). Out of these analogs, treatment with **5l** resulted in the strongest effects that matched the outcomes observed for clinically tested KZR-616 and ONX-0914 (Figure 4). These three compounds most potently decreased the secretion of IL-6, IL-10, and TNF-α, the cytokines that collectively define a pro-inflammatory profile. This inflammatory program originates predominantly from innate immune cells such as monocytes and macrophages responding to pathogen-associated stimuli (e.g., LPS) but is further amplified and sustained by activated T cells, B cells, NK cells, and stromal cells during prolonged inflammation.^86^ Importantly, dysregulated or chronic production of these cytokines, particularly TNF-α, IL-1, and IL-6, is a central pathogenic feature of autoimmune diseases, where their persistent signaling promotes leukocyte recruitment, maintains immune activation, and contributes directly to tissue damage.^87,88^ Inhibition of the iCP disrupts activation of key inflammatory signaling pathways, such as NF-κB, resulting in reduced transcription and secretion of these mediators. Thus, the observed decrease in IL-1, IL-6, IL-8, and TNF-α secretion reflects a suppression of the cellular processes that initiate and sustain pathological inflammation. Altogether, these data reconfirm that inhibition of at least two iCP subunits is imperative to achieve a pharmacologically meaningful attenuation of pro-inflammatory signaling, as well as highlight the potential of novel α-aminoboronic acids as anti-inflammatory agents.

For compound **5l**, as well as bortezomib, the dose-dependent effects on cytokine release from LPS-stimulated PBMCs were also determined (Figure S12). As shown by the dose–response curves, **5l** and bortezomib had very comparable effects, with the latter exhibiting slightly greater efficacy in reducing TNF-α and IL-10 levels. To further confirm that the reduction in inflammatory cytokine release was a consequence of proteasome inhibition in intact PBMCs, a cell-based Proteasome-Glo assay was performed with **5l** and bortezomib (Figure 5). Both compounds inhibited the β5 activity of PMBCs to a similar extent with the IC_50_ values in low nanomolar range. The similar potencies are consistent with the structural similarity between the two compounds. These findings confirm that the inhibition of cytokine release from LPS-stimulated PBMCs is most likely a direct result of immuno/proteasome inhibition.

**Figure 5:**
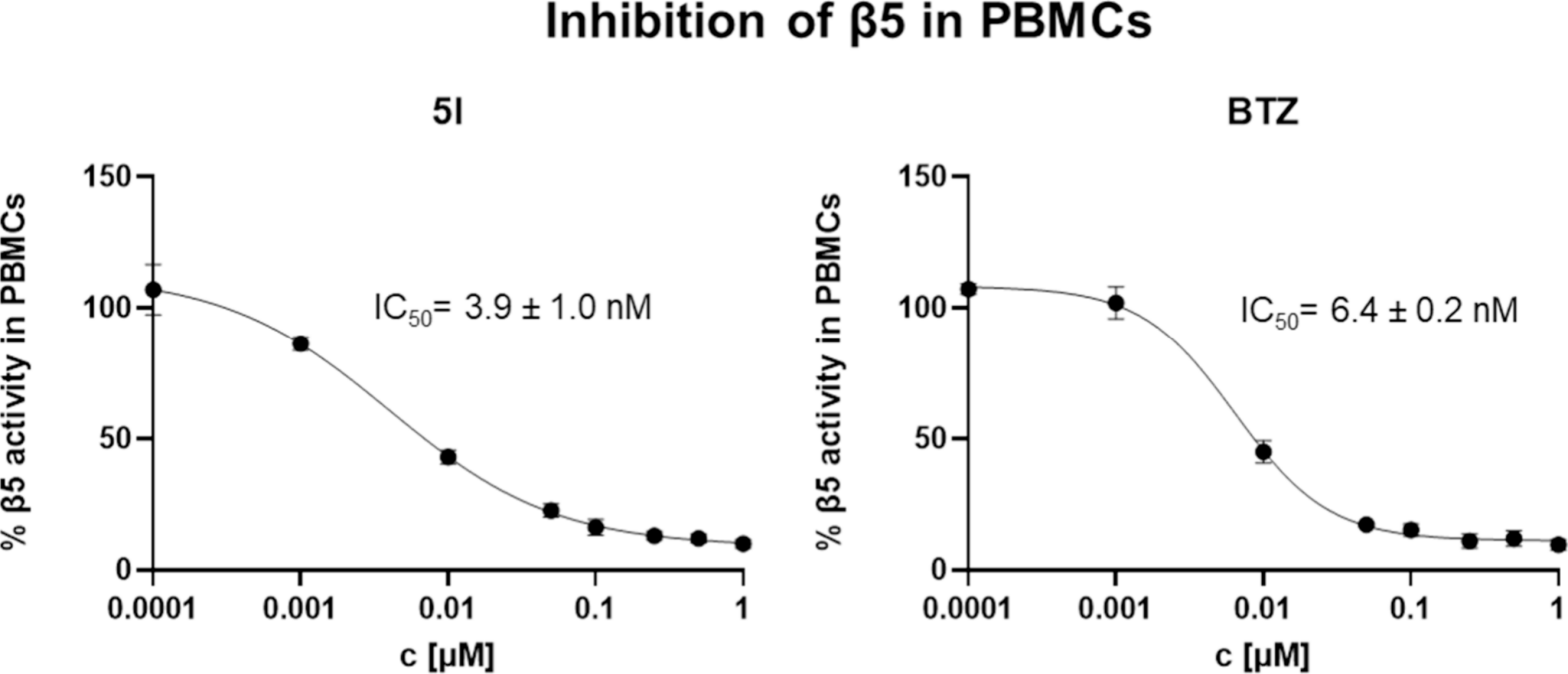
Inhibition of β5 activity of PBMCs by compounds **5l** and bortezomib, determined using the Proteasome-Glo assay. PBMCs were treated with the inhibitors at various concentrations for 6 h. Data are presented as mean ± SD from three independent experiments (*N* = 3).

Next, we conducted preliminary *in vitro* pharmacokinetic studies to assess the basic ADME properties of compound **5l**. We evaluated the kinetic solubility, microsomal stability, plasma protein binding (PPB), and permeability of **5l**, using bortezomib as a reference. The results are summarized in Tables S3–S6 and Figure S13. Overall, both analogs displayed similar properties, showing high solubility (>200 μM), good parallel artificial membrane permeability (P_e_ = 15.8 × 10^6^ cm/s), and similar PPB (91% for **5l** and 93% for bortezomib). Permeability was evaluated also using Caco-2 cells, in which bortezomib showed a slightly lower efflux ratio and marginally higher permeability (bortezomib: P_app_ (A-B) = 2.8 ± 0.1 × 10^–6^ cm/s, P_app_ (B-A) = 20.7 ± 0.1 × 10^–6^ cm/s; **5l**: P_app_ (A-B) = 2.0 ± 0.2 × 10^–6^ cm/s, P_app_ (B-A) = 17.1 ± 1 × 10^–6^ cm/s). On the other hand, **5l** demonstrated higher microsomal stability (Figure S13). Since bortezomib is an established, clinically used drug with well-characterized pharmacokinetic and ADME properties, these findings further support compound **5l** as a promising candidate for continued pharmacological evaluation.

## Conclusions

In this work, we utilized a straightforward synthetic approach to prepare a focused series of boro dipeptides with distinct and unconventional P1 side chains that are not typically used in studies of substrate preferences.^60^ The compounds showed unique immuno/proteasome inhibition profiles, such as β1i/β5i-selectivity (compounds **5b**–**5d**), preference for β5c/β5i and β1i/β2i subunits (compound **5h**), and a bortezomib-like profile favoring the β1 and β5 subunits of both proteasomes (compounds **5j** and **5l**), thus representing an important contribution to novel chemical matter targeting cCP and iCP. From a pharmacological standpoint, the bortezomib analog **5l** proved to be the most interesting. Although it did not reach the anticancer activity of bortezomib, it showed a very potent inhibition of cytokine production, without affecting the viability of PBMCs. Of note, the anti-inflammatory activity of **5l** was comparable to that of zetomipzomib (KZR-616). Despite the limited number of compounds and a basic evaluation of their cellular effects, we believe that the results represent a solid basis for further tailoring of α-aminoboronic acid residues to yield compounds with improved inhibition and subunit-specific profiles. The advanced α-aminoboronic acid–based derivatives might have important therapeutic potential, especially as anticancer and anti-inflammatory agents, as well as prove useful in further deciphering of the (pato)­physiological functions of different cCP and iCP subunits.

## Experimental Section

### General Chemistry Remarks

All reagents were purchased from commercial vendors (Merck-Sigma-Aldrich, TCI, Acros Organics, Abcr, BLDPharm, STREM, Alfa Aesar) and were used as supplied unless noted otherwise. 1-Diol resin was purchased from Iris Biotech GmbH. Syringes for solid-phase peptide syntheses were purchased from Carl Roth. Compound **1f** was purchased from Sigma-Aldrich. Commercial samples of carfilzomib, bortezomib, M3258, KZR-504, ML604440, Lu-002i, KZR-616, and ONX-0914 were purchased from MedChemExpress. DPLG3 was synthesized in house according to the literature procedure.^89^ Reactions were monitored using analytical thin-layer chromatography (TLC) on silica gel 60 F254 Al plates (Merck) and spots were detected under UV light (254 or 365 nm) or visualized by ninhydrin or phosphomolybdate staining. ^1^H, ^11^B, ^13^C and ^19^F nuclear magnetic resonance spectra were recorded at the University of Ljubljana, Faculty of Pharmacy with a Bruker AVANCE III 400 at 400, 128, 101, and 376 MHz, respectively, using acetone-*d*_6_, MeOD or DMSO-*d*_6_ as solvents at 295 K. NMR spectra were processed and analyzed in MestReNova. All chemical shifts are reported in ppm, with ^1^H and ^13^C resonances referenced to the residual nondeuterated solvent signal as internal standard. Coupling constants *J* are reported in Hz. Please note that the resonances for boron-bound carbon nuclei were broadened and often suppressed in ^13^C spectra. For final compounds **5**, the ppms of the major diastereomer are provided (the diastereomeric ratios between (*R*,*S*)-**5** diastereomer and (*S*,*S*)-**5** diastereomer were ranging from 82:18 to 99:1). Attenuated total reflectance (ATR) infrared spectra were recorded on a FT-IR Thermo Nicolet spectrometer. High-resolution mass measurements were recorded on Thermo Scientific Q Exactive Plus LC-MS/MS mass spectrometer (Thermo Fisher Scientific). HPLC purity with concurrent HRMS confirmation was performed on UltiMate 3000 UHPLC system coupled to a Q-Exactive Plus Orbitrap mass spectrometer (Thermo Fisher Scientific, USA) Chromatographic separation was achieved on an Acquity UPLC BEH C18 column (50 mm × 2.1 mm, 1.7 μm; Waters, USA) maintained at 40 °C. The autosampler compartment was set to 10 °C, with an injection volume of 5.00 μL. The mobile phase consisted of water/MeCN/formic acid (950/50/1) as solvent A and water/MeCN/formic acid (50/950/1) as solvent B, using the following gradient: 0–1.0 min, 0% B; 1.0–8.0 min, 0%–100% B; 8.0–10.0 min, 100% B; 10.0–10.1 min, 100%–0% B; 10.1–12.0 min, 0% B. The flow rate was kept constant at 0.50 mL/min throughout the analysis. The HESI ion source on Q-Exactive Plus Orbitrap mass spectrometer switched between positive and negative ionization mode with the following settings: sheath gas flow rate, 25 units; auxiliary gas flow rate, 10 units; capillary temperature, 350 °C; spray voltage, 3.5 kV; and S-lens RF level, 50. Simultaneously, UV absorption was monitored at 255 nm. Instrument control, data acquisition, and quantification were conducted using Xcalibur software (Thermo Fisher Scientific). All compounds that were evaluated in biological assays were >95% pure by HPLC analysis.

### Synthesis Details

For this work, our recently discovered synthetic methodology toward α-aminoboronic acids **4** was used.^66^ Apart from **2f**, **3f**, and **4f**, all precursors for the synthesis of α-aminoboronic acids **4** have been fully characterized previously.^66^ For the sake of clarity, the synthetic methodology is briefly condensed below and general procedures for each step are provided.

### General Procedure for the Synthesis of Primary Trifluoroborate Iminiums (pTIMs) **2a**–**2h**

In an oven-dried flask under argon atmosphere, KAT **1** (1 equiv) and NH_4_Cl (5 equiv) were dissolved in dry MeOH (0.08 M) and stirred at 40 °C. The reaction was typically complete in 3–6 h for aromatic substrates **1a**–**1h** and 1 h for aliphatic substrates **1i**–**1l**. When all starting material had been consumed (tracked by ^1^H NMR of the crude reaction mixture), the solvent was evaporated under reduced pressure and dry residue was suspended in EtOAc. The suspension was filtered, washed with EtOAc, and the filtrate was evaporated under reduced pressure to afford pTIMs **2** which required no additional purification.

### ((3-(Ethoxycarbonyl)­phenyl)­(iminio)­methyl)­trifluoroborate (**2f**)

Synthesized from potassium 3-ethoxycarbonyl­benzoyl­trifluoroborate (940 mg, 3.31 mmol) and NH_4_Cl (885 mg, 16.55 mmol) in 6 h according to the General procedure, to afford the title compound as a pale-yellow solid (796 mg, 98% yield). ^1^H NMR (400 MHz, Acetone-*d*_6_) δ 1.38 (t, *J* = 7.1 Hz, 3H, CH_3_), 4.40 (q, *J* = 7.1 Hz, 2H, CH_2_), 7.78 (t, *J* = 7.8 Hz, 1H, Ar-H), 8.34 (dt, *J* = 7.8, 1.3 Hz, 1H, Ar-H), 8.44 (ddd, *J* = 7.8, 1.9, 1.1 Hz, 1H, Ar-H), 8.83 (t, *J* = 1.6 Hz, 1H, Ar-H), 10.92 (br t, *J* = 66.8 Hz, 1H, NH_2a_^+^), 11.35 (br t, *J* = 62.0 Hz, 1H, NH_2b_^+^). ^11^B NMR (128 MHz, Acetone-*d*_6_) δ 0.04 (q, *J* = 38.7 Hz). ^13^C NMR (101 MHz, Acetone-*d*_6_) δ 14.50, 62.06, 130.39, 131.49, 132.39, 134.05, 135.29, 135.88, 165.74, 207 (br m, extracted from ^1^H–^13^C HMBC spectrum). ^19^F NMR (376 MHz, Acetone-*d*_6_) δ −146.02 (dd, *J* = 76.9, 37.9 Hz). IR (ν/cm^–1^, ATR) ν_max._ = 823, 866, 903, 928, 991, 1007, 1026, 1051, 1076, 1111, 1152, 1281, 1611, 1664, 1708, 3206, 3321. HRMS (ESI^–^): calc. for C_10_H_10_BNO_2_F_3_ [M – H]^−^: 244.0762, found: 244.0761.

### General Procedure for the Synthesis of Aromatic Primary Trifluoborate Ammoniums (pTAMs) **3a**–**3h**

pTIM **2** was dissolved in MeOH (containing 2% V/V H_2_O, 0.1 M) in a flask and [(*R,R*)-TethTsDpen-RuCl] (2 mol %) was added to the solution. No oven-dried glassware was used and the reaction was set up in air without flushing the flask with argon or nitrogen. The flask was transferred to a hydrogenation reactor. The reactor was purged with H_2_ five times, after which the H_2_ pressure was set to 20 bar and the reaction mixture was left to stir at rt for 20 h. Then, the pressure was carefully released, the solvent evaporated under reduced pressure, and the crude dry residue was analyzed by NMR and HPLC to determine the conversion and the enantiomeric ratio. The crude product was suspended in CH_2_Cl_2_ (0.5 mL), filtered, and washed with *n*-hexane (2 × 5 mL) to afford the product which required no additional purification. Exceptions from the General procedure: **3e** (concentration 0.05 M, reaction time 44 h), **3g** (concentration 0.05 M), **3h** (concentration 0.017 M). The enantiomeric ratios (R/S) were as follows: **3a** (93:7), **3b** (91:9), **3c** (97:3), **3d** (93:7), **3e** (90:10), **3f** (n.d.), **3g** (92:8), **3h** (98:2).

### General Procedure for the Synthesis of Aliphatic pTAMs **3i**–**3l**

The reaction procedure followed the General procedure for aromatic pTAMs in most steps. Importantly, [(*S,S*)-TethTsDpen-RuCl] (4 mol %) was used here since we previously^66^ discovered that the (*S,S*)-catalyst affords the desired (*R*)-pTAMs **3** from aliphatic pTIMs **2**, which is in contrast to their aromatic counterparts which require the (*R*,*R*)-catalyst. Also, it was required to prolong the reaction time to 72 h. The crude product was suspended in CH_2_Cl_2_ (0.5 mL), filtered, and washed with CH_2_Cl_2_ (1 mL) and *n*-hexane (5 mL) to afford the product which required no additional purification. The enantiomeric ratios (*R*/*S*) were as follows: **3i** (94:6), **3j** (93:7), **3k** (96:4), **3l** (99:1).

### (Ammonio­(3-(ethoxycarbonyl)­phenyl)­methyl)­trifluoroborate (**3f**)

Synthesized from **2f** (98 mg, 0.4 mmol) according to the General procedure to afford the title compound as a pale-yellow solid (94 mg, 95% yield). ^1^H NMR (400 MHz, MeOD) δ 1.38 (t, *J* = 7.1 Hz, 3H, CH_3_), 3.34 – 3.39 (m, 1H, CH), 4.36 (q, *J* = 7.1 Hz, 2H, CH_2_), 7.41 (t, *J* = 7.7 Hz, 1H, Ar-H), 7.55 (dt, *J* = 7.7, 1.5 Hz, 1H, Ar-H), 7.87 (dt, *J* = 7.7, 1.4 Hz, 1H, Ar-H), 8.00 (t, *J* = 1.8 Hz, 1H, Ar-H). ^11^B NMR (128 MHz, MeOD) δ 2.51 (m). ^13^C NMR (101 MHz, MeOD) δ 14.59, 50.94 (br m), 62.11, 128.27, 128.84, 129.28, 131.46, 132.86, 142.37, 168.27. ^19^F NMR (376 MHz, MeOD) δ −151.42. IR (ν/cm^–1^, ATR) ν_max._ = 720, 757, 864, 913, 945, 1008, 1133, 1201, 1252, 1287, 1367, 1447, 1507, 1601, 1698, 2987, 3251. HRMS (ESI^–^): calc. for C_10_H_12_BNO_2_F_3_ [M – H]^−^: 246.0919, found: 246.0918.

### General Procedure for the Synthesis of α-Aminoboronic Acids **4**

pTAM **3** was dissolved in MeOH (0.05 M). To this solution, HCl (2 M aq, 4 equiv) and HMDSO (3 equiv) were added and the solution was left to stir at rt until the reaction was complete (monitored by ^11^B NMR of crude reaction mixture – a 200 μL sample was diluted with 300 μL MeOD), typically from 2 to 4 h. Then, the volatiles were removed under reduced pressure, whereby the flask was not heated over 30 °C. The residue was evaporated to dryness on an oil pump (0.02 bar, rt, 30 min), to afford the α-aminoboronic acids **4** as hydrochloride salts. No isolation procedure was required since all reagents and side products are volatile compounds and the reactions proceeded in a fully chemoselective fashion.

### Borono­(3-(ethoxycarbonyl)­phenyl)­methanaminium Chloride (**4f**)

Synthesized from **3f** (25 mg, 0.1 mmol) according to the General procedure to afford the title compound as a white foam-like solid (25 mg, 96% yield). ^1^H NMR (400 MHz, MeOD) δ 1.40 (t, *J* = 7.1 Hz, 3H, CH_3_), 4.12 (s, 1H, CH), 4.39 (q, *J* = 7.1 Hz, 2H, CH_2_), 7.57 (t, *J* = 7.6 Hz, 1H, Ar-H), 7.68 (d, *J* = 7.4 Hz, 1H, Ar-H), 8.05 (d, *J* = 7.5 Hz, 1H, Ar-H), 8.09 – 8.13 (m, 1H, Ar-H). ^11^B NMR (128 MHz, MeOD) δ 28.26. ^13^C NMR (101 MHz, MeOD) δ 14.58, 45 (br m, extracted from ^1^H–^13^C HSQC spectrum), 62.45, 130.78 (2C), 131.15, 132.86, 134.95, 136.83, 167.34. HRMS (ESI^+^): calc. for C_10_H_15_BNO_4_ [M-Cl]^+^: 224.1089, found: 224.1088.

### General Procedure for the Synthesis of P1-Derivatized Bortezomib Analogs **5**

Since pTAMs **3** are more stable and easier to handle than α-aminoboronic acids **4**, the latter were prepared *in situ* from **3** and directly used in the solid-phase peptide synthesis protocol. pTAM **3** was subjected to the ‘General procedure for the synthesis of α-aminoboronic acids’ described above, so the α-aminoboronic acid **4** could be used directly after it was prepared (quantitative yield assumed).

### Preparation of the 1-Diol Resin

In a 5 mL PP syringe equipped with a PE frit, 1-diol resin (167 mg, loading 0.6 mmol/g binding capacity, 1 equiv) was suspended in CH_2_Cl_2_ (2 mL) and shaken for 30 min, allowing the resin to swell, then the syringe was emptied of the solvent and dried by vacuum filtration.

**Step 1.** After thorough drying, the α-aminoboronic acid **4** (0.1 mmol) was dissolved in DMF (2 mL), added to the syringe with the swollen resin, and shaken for 16 h.

**Step 2.** The syringe was washed with DMF (6 × 1.5 mL). In a separate flask on an ice bath, Fmoc-l-Phe-OH (116 mg, 3 equiv), HATU (114 mg, 3 equiv), and DIPEA (87 μL, 5 equiv) were dissolved in DMF (2 mL). The yellow solution was stirred in the flask at 0 °C for 5 min and then transferred to the syringe, which was shaken for 2 h.

**Step 3.** The syringe was emptied of the solvent and washed with DMF (3 × 1.5 mL), CH_2_Cl_2_ (3 × 1.5 mL), and again with DMF (3 × 1.5 mL). Piperidine (10% in DMF, 1.5 mL) was added to the syringe, which was shaken for 10 min, emptied, followed by repeating the procedure and shaking for 5 min. The syringe was washed with DMF (3 × 1.5 mL), CH_2_Cl_2_ (3 × 1.5 mL), and again with DMF (3 × 1.5 mL).

**Step 4.** In a separate flask, pyrazine-2-carboxylic acid (37 mg, 3 equiv), HATU (114 mg, 3 equiv), and DIPEA (70 μL, 4 equiv) were dissolved in DMF (2 mL). The red solution was stirred in the flask at 0 °C for 5 min and then transferred to the syringe, which was shaken for 2 h.

**Step 5.** The syringe was emptied of the solvent and washed with DMF (6 × 1.5 mL) and CH_2_Cl_2_ (9 × 1.5 mL). Then, CH_2_Cl_2_:MeOH:H_2_O = 5:4:1 (2 mL) was added and the syringe was shaken for 30 min. The procedure was repeated three times. The filtrate was collected and the solvent was removed under reduced pressure. While the crude products did not contain significant impurities based on NMR spectra analysis, they were further purified for biological testing.

### Purification Procedure for Final Compounds **5**

All products were purified on a Biotage Isolera One, using a Biotage Sfär C18 D – Duo 100 Å 30 μm reverse-phase column. Mobile phase consisted of two components: 0.1% TFA and MeCN (gradient 40% MeCN to 80% MeCN over 20 min), flow rate = 12 mL/min. The appropriate fractions were collected and evaporated under reduced pressure. The dry residue was dissolved in 0.5 mL MeCN or MeOH, to which water (1 mL) was added and a white precipitate formed. The precipitate was filtered off to afford a compound that was used in testing.

### ((*R*)-Phenyl­((*S*)-3-Phenyl-2-(pyrazine-2-carboxamido)­propanamido)­methyl)­boronic Acid (**5a**)

Synthesized according to the General procedure from **3a** (17.5 mg, 0.1 mmol) to afford the title compound as a white solid (14 mg, 35% yield). The d.r. between (*R*,*S*) and (*S*,*S*) was 87:13. ^1^H NMR (400 MHz, MeOD) δ 3.36 (dd, *J* = 13.9, 8.5 Hz, 1H, CH_2a_), 3.45 (dd, *J* = 13.8, 6.4 Hz, 1H, CH_2b_), 3.74 (s, 1H, CH-B), 5.19 (ddd, *J* = 8.5, 6.4, 1.2 Hz, 1H, CH-Bn), 7.11 – 7.18 (m, 3H, Ar-H), 7.21 – 7.27 (m, 3H, Ar-H), 7.28 – 7.38 (m, 4H, Ar-H), 8.69 (dd, *J* = 2.5, 1.5 Hz, 1H, Pyz-H), 8.78 (d, *J* = 2.5 Hz, 1H, Pyz-H), 9.19 (d, *J* = 1.5 Hz, 1H, Pyz-H). ^13^C NMR (101 MHz, MeOD) δ 37.83, 52.79, 54.21 (br m), 126.62, 127.18, 128.34, 129.05, 129.80, 130.49, 137.20, 142.10, 144.80, 144.90, 145.68, 148.81, 165.61, 178.80. HRMS (ESI^–^): calc. for C_21_H_20_BN_4_O_4_ [M – H]^−^: 403.1583, found: 403.1581.

### ((*R*)-((*S*)-3-Phenyl-2-(pyrazine-2-carboxamido)­propanamido)­(*p*-tolyl)­methyl)­boronic Acid (**5b**)

Synthesized according to the General procedure from **3b** (19 mg, 0.1 mmol) to afford the title compound as a white solid (16 mg, 38% yield). The d.r. between (*R*,*S*) and (*S*,*S*) was 90:10. ^1^H NMR (400 MHz, MeOD) δ 2.28 (s, 3H, CH_3_), 3.33 – 3.38 (m, 1H, CH_2a_), 3.43 (dd, *J* = 13.8, 6.4 Hz, 1H, CH_2b_), 3.70 (s, 1H, CH-B), 5.13 – 5.20 (m, 1H, CH-Bn), 6.98 – 7.04 (m, 2H, Ar-H), 7.04 – 7.10 (m, 2H, Ar-H), 7.26 – 7.37 (m, 5H, Ar-H), 8.69 (dd, *J* = 2.5, 1.5 Hz, 1H, Pyz-H), 8.78 (d, *J* = 2.5 Hz, 1H, Pyz-H), 9.19 (d, *J* = 1.5 Hz, 1H, Pyz-H). ^13^C NMR (101 MHz, MeOD) δ 21.06, 37.83, 52.68, 53.92 (br m), 127.19, 128.34, 129.70, 129.80, 130.49, 136.23, 137.17, 138.80, 144.80, 144.89, 145.68, 148.81, 165.59, 178.75. HRMS (ESI^–^): calc. for C_22_H_22_BN_4_O_4_ [M – H]^−^: 417.1740, found: 417.1737.

### ((*R*)-(4-Fluorophenyl)­((*S*)-3-phenyl-2-(pyrazine-2-carboxamido)­propanamido)­methyl)­boronic Acid (**5c**)

Synthesized according to the General procedure from **3c** (19 mg, 0.1 mmol) to afford the title compound as a white solid (20 mg, 47% yield). The d.r. between (*R*,*S*) and (*S*,*S*) was 94:6. ^1^H NMR (400 MHz, MeOD) δ 3.35 – 3.39 (m, 1H, CH_2a_), 3.41 – 3.46 (m, 1H, CH_2b_), 3.72 (s, 1H, CH-B), 5.16 (ddd, *J* = 8.0, 6.6, 1.0 Hz, 1H, CH-Bn), 6.95 – 7.00 (m, 2H, Ar-H), 7.11 – 7.16 (m, 2H, Ar-H), 7.23 – 7.27 (m, 1H, Ar-H), 7.29 – 7.37 (m, 4H, Ar-H), 8.70 (br s, 1H, Pyz-H), 8.80 (br s, 1H, Pyz-H), 9.21 (br s, 1H, Pyz-H). ^13^C NMR (101 MHz, MeOD) δ 37.73, 52.77, 115.64 (d, ^2^ *J*_CF_ = 21.3 Hz), 128.38, 128.85, 128.93, 129.82, 130.48, 137.13, 137.92 (d, ^3^ *J*_CF_ = 2.9 Hz), 144.84, 144.91, 148.84, 162.69 (d, ^1^ *J*_CF_ = 241.8 Hz), 165.67, 179.07. HRMS (ESI^–^): calc. for C_21_H_19_BN_4_O_4_F [M – H]^−^: 421.1489, found: 421.1485.

### ((*R*)-(4-Chlorophenyl)­((*S*)-3-phenyl-2-(pyrazine-2-carboxamido)­propanamido)­methyl)­boronic Acid (**5d**)

Synthesized according to the General procedure from **3d** (21 mg, 0.1 mmol) to afford the title compound as a white solid (18 mg, 41% yield). The d.r. between (*R*,*S*) and (*S*,*S*) was 88:12. ^1^H NMR (400 MHz, MeOD) δ 3.36 – 3.39 (m, 1H, CH_2a_), 3.44 (dd, *J* = 13.8, 6.4 Hz, 1H, CH_2b_), 3.72 (s, 1H CH-B), 5.16 (ddd, *J* = 7.7, 6.5, 1.1 Hz, 1H, CH-Bn), 7.08 – 7.13 (m, 2H, Ar-H), 7.22 – 7.26 (m, 3H, Ar-H), 7.29 – 7.38 (m, 4H, Ar-H), 8.69 (dd, *J* = 2.6, 1.5 Hz, 1H, Pyz-H), 8.78 (d, *J* = 2.5 Hz, 1H, Pyz-H), 9.20 (d, *J* = 1.5 Hz, 1H, Pyz-H). ^13^C NMR (101 MHz, MeOD) δ 37.75, 56.16, 128.37, 128.74, 129.03, 129.52, 129.82, 130.48, 137.16, 137.89, 144.80, 144.90, 145.65, 148.84, 165.65, 179.06. HRMS (ESI^–^): calc. for C_21_H_19_BN_4_O_4_Cl [M – H]^−^: 437.1193, found: 437.1191.

### ((*R*)-(4-Methoxyphenyl)­((*S*)-3-phenyl-2-(pyrazine-2-carboxamido)­propanamido)­methyl)­boronic Acid (**5e**)

Synthesized according to the General procedure from **3e** (20.5 mg, 0.1 mmol) to afford the title compound as a white solid (15 mg, 35% yield). The d.r. between (*R*,*S*) and (*S*,*S*) was 84:16. ^1^H NMR (400 MHz, MeOD) δ 3.33 – 3.37 (m, 1H, CH_2a_), 3.43 (dd, *J* = 13.8, 6.3 Hz, 1H, CH_2b_), 3.68 (s, 1H, CH-B), 3.75 (s, 3H, CH_3_), 5.16 (app t, *J* = 7.4 Hz, 1H, CH-Bn), 6.79 – 6.84 (m, 2H, Ar-H), 7.03 – 7.08 (m, 2H, Ar-H), 7.20 – 7.27 (m, 1H, Ar-H), 7.28 – 7.40 (m, 4H, Ar-H), 8.69 (dd, *J* = 2.5, 1.5 Hz, 1H, Pyz-H), 8.78 (d, *J* = 2.5 Hz, 1H, Pyz-H), 9.19 (d, *J* = 1.5 Hz, 1H, Pyz-H). ^13^C NMR (101 MHz, MeOD) δ 39.75, 52.21, 55.63, 114.67, 128.44, 129.48, 129.60, 129.80, 130.42, 130.56, 131.79, 144.69, 144.82, 145.74, 148.82, 168.02, 179.61. HRMS (ESI^–^): calc. for C_22_H_22_BN_4_O_5_ [M – H]^−^: 433.1689, found: 433.1685.

### ((*R*)-(3-(Ethoxycarbonyl)­phenyl)­((*S*)-3-phenyl-2-(pyrazine-2-carboxamido)­propanamido)­methyl)­boronic Acid (**5f**)

Synthesized according to the General procedure from **3f** (25 mg, 0.1 mmol) to afford the title compound as a white solid (20 mg, 42% yield). The d.r. between (*R*,*S*) and (*S*,*S*) was >99:1. ^1^H NMR (400 MHz, MeOD) δ 1.36 (t, *J* = 7.1 Hz, 3H, CH_3_), 3.42 – 3.50 (m, 2H, CH_2_-Ph), 3.82 (s, 1H, CH-B), 4.34 (q, *J* = 7.1 Hz, 2H, CH_2_CH_3_), 5.14 – 5.23 (m, 1H, CH-Bn), 7.23 – 7.27 (m, 1H, Ar-H), 7.29 – 7.34 (m, 2H, Ar-H), 7.34 – 7.40 (m, 4H, Ar-H), 7.76 – 7.85 (m, 2H, Ar-H), 8.69 (dd, *J* = 2.5, 1.5 Hz, 1H, Pyz-H), 8.79 (d, *J* = 2.5 Hz, 1H, Pyz-H), 9.21 (d, *J* = 1.5 Hz, 1H, Pyz-H). ^13^C NMR (101 MHz, MeOD) δ 14.62, 37.71, 52.79, 62.06, 127.79, 128.02, 128.37, 129.27, 129.84, 130.46, 131.44, 131.88, 137.17, 142.91, 144.83, 144.97, 145.72, 148.82, 165.68, 168.25, 179.43. HRMS (ESI^–^): calc. for C_24_H_24_BN_4_O_6_ [M – H]^−^: 475.1794, found: 475.1791.

### ((*R*)-Benzo­[*d*]­[1,3]­dioxol-5-yl­((*S*)-3-phenyl-2-(pyrazine-2-carboxamido)­propanamido)­methyl)­boronic Acid (**5g**)

Synthesized according to the General procedure from **3g** (22 mg, 0.1 mmol) to afford the title compound as a white solid (16 mg, 36% yield). The d.r. between (*R*,*S*) and (*S*,*S*) was 83:17. ^1^H NMR (400 MHz, MeOD) δ 3.34 – 3.37 (m, 1H, CH_2a_-Ph), 3.43 (dd, *J* = 13.8, 6.4 Hz, 1H, CH_2b_-Ph), 3.66 (s, 1H CH-B), 5.11 – 5.19 (m, 1H CH-Bn), 5.88 (s, 2H O–CH_2_-O), 6.57 – 6.62 (m, 1H, Ar-H), 6.64 – 6.72 (m, 2H, Ar-H), 7.22 – 7.27 (m, 1H, Ar-H), 7.28 – 7.37 (m, 4H, Ar-H), 8.69 (br s, 1H, Pyz-H), 8.80 (br s, 1H, Pyz-H), 9.21 (br s, 1H, Pyz-H). ^13^C NMR (101 MHz, MeOD) δ 37.72, 52.71, 102.06, 108.06, 108.74, 120.25, 128.37, 129.82, 129.98, 130.48, 130.54, 135.84, 137.16, 144.88, 147.17, 148.78, 149.08, 165.65, 178.81. HRMS (ESI^–^): calc. for C_22_H_20_BN_4_O_6_ [M – H]^−^: 447.1481, found: 447.1478.

### ((*R*)-[1,1′-Biphenyl]-4-yl­((*S*)-3-phenyl-2-(pyrazine-2-carboxamido)­propanamido)­methyl)­boronic Acid (**5h**)

Synthesized according to the General procedure from **3h** (25 mg, 0.1 mmol) to afford the title compound as a white solid (18 mg, 38% yield). The d.r. between (*R*,*S*) and (*S*,*S*) was 84:16. ^1^H NMR (400 MHz, MeOD) δ 3.36 – 3.40 (m, 1H, CH_2a_), 3.48 (dd, *J* = 14.2, 6.5 Hz, 1H, CH_2b_), 3.82 (s, 1H, CH-B), 5.22 (app t, *J* = 7.5 Hz, 1H, CH-Bn), 7.23 – 7.37 (m, 6H, Ar-H), 7.37 – 7.45 (m, 4H, Ar-H), 7.51 – 7.63 (m, 4H, Ar-H), 8.72 (br s, 1H, Pyz-H), 8.82 (br s, 1H, Pyz-H), 9.25 (br s, 1H, Pyz-H). ^13^C NMR (101 MHz, MeOD) δ 37.83, 56.17, 127.60, 127.69, 127.74, 128.03, 128.37, 129.59, 129.80, 129.83, 130.50, 130.60, 137.20, 142.35, 144.82, 144.92, 145.70, 148.83, 165.65, 178.98. HRMS (ESI^–^): calc. for C_27_H_24_BN_4_O_4_ [M – H]^−^: 479.1896, found: 479.1792.

### ((*R*)-2-Phenyl-1-((*S*)-3-phenyl-2-(pyrazine-2-carboxamido)­propanamido)­ethyl)­boronic Acid (**5i**)

Synthesized according to the General procedure from **3i** (19 mg, 0.1 mmol) to afford the title compound as a white solid (21 mg, 50% yield). The d.r. between (*R*,*S*) and (*S*,*S*) was 82:18. ^1^H NMR (400 MHz, MeOD) δ 2.41 (dd, *J* = 13.9, 9.9 Hz, 1H, CH_2a_), 2.78 (dd, *J* = 13.9, 5.5 Hz, 1H, CH_2b_), 2.84 – 2.90 (m, 1H, CH-B), 3.20 (dd, *J* = 13.6, 7.9 Hz, 1H, CH_2a_), 3.25 – 3.29 (m, 1H, CH_2b_), 5.01 (t, *J* = 7.7 Hz, 1H, CH-Bn), 6.97 – 7.04 (m, 2H, Ar-H), 7.08 – 7.13 (m, 1H, Ar-H), 7.14 – 7.23 (m, 3H, Ar-H), 7.27 – 7.30 (m, 1H, Ar-H), 7.31 – 7.35 (m, 3H, Ar-H), 8.70 (br s, 1H, Pyz-H), 8.81 (br s, 1H, Pyz-H), 9.19 (br s, 1H, Pyz-H). ^13^C NMR (101 MHz, MeOD) δ 38.04, 38.57, 52.97, 127.02, 128.39, 129.36, 129.84, 129.96, 130.57, 130.58, 137.14, 141.84, 144.87, 144.90, 148.96, 165.17, 177.27. HRMS (ESI^–^): calc. for C_22_H_22_BN_4_O_4_ [M – H]^−^: 417.1740, found: 417.1736.

### ((*R*)-1-((*S*)-3-phenyl-2-(pyrazine-2-carboxamido)­propanamido)­propyl)­boronic Acid (**5j**)

Synthesized according to the General procedure from **3j** (13 mg, 0.1 mmol) to afford the title compound as a white solid (16 mg, 45% yield). The d.r. between (*R*,*S*) and (*S*,*S*) was 86:14. ^1^H NMR (400 MHz, MeOD) δ 0.84 (t, *J* = 7.4 Hz, 3H, CH_3_), 1.25 – 1.52 (m, 2H, CH_2_-CH_3_), 2.50 (t, *J* = 7.4 Hz, 1H, CH-B), 3.21 – 3.30 (m, 2H, CH_2_-Ph), 5.05 (t, *J* = 7.6 Hz, 1H, CH-Bn), 7.20 – 7.24 (m, 1H, Ar-H), 7.26 – 7.32 (m, 4H, Ar-H), 8.71 (s, 1H, Pyz-H), 8.83 (s, 1H, Pyz-H), 9.21 (s, 1H, Pyz-H). ^13^C NMR (101 MHz, MeOD) δ 12.48, 24.61, 38.43, 52.87, 128.28, 129.73, 130.48, 137.09, 144.81, 144.90, 148.88, 148.93, 162.27, 177.09. HRMS (ESI^–^): calc. for C_17_H_20_BN_4_O_4_ [M – H]^−^: 355.1583, found: 355.1583.

### ((*R*)-Cyclopropyl­((*S*)-3-phenyl-2-(pyrazine-2-carboxamido)­propanamido)­methyl)­boronic Acid (**5k**)

Synthesized according to the General procedure from **3k** (14 mg, 0.1 mmol) to afford the title compound as a white solid (19 mg, 52% yield). The d.r. between (*R*,*S*) and (*S*,*S*) was 88:12. ^1^H NMR (400 MHz, MeOD) δ 0.05 – 0.17 (m, 2H, *c*Pr–CH_2_), 0.35 – 0.52 (m, 2H, *c*Pr–CH_2_), 0.69 – 0.80 (m, 1H, *c*Pr–CH), 1.78 (d, *J* = 10.4 Hz, 1H, CH-B), 3.25 (dd, *J* = 13.7, 8.0 Hz, 1H, CH_2a_), 3.33 – 3.36 (m, 1H, CH_2b_), 5.09 (t, *J* = 7.5 Hz, 1H, CH-Bn), 7.19 – 7.24 (m, 1H, Ar-H), 7.25 – 7.34 (m, 4H, Ar-H), 8.69 (dd, *J* = 2.5, 1.5 Hz, 1H, Pyz-H), 8.79 (d, *J* = 2.5 Hz, 1H, Pyz-H), 9.16 (d, *J* = 1.5 Hz, 1H, Pyz-H). ^13^C NMR (101 MHz, MeOD) δ 4.37, 5.12, 12.13, 38.42, 52.55, 54.75 (br m), 128.28, 129.74, 130.53, 137.06, 144.81, 144.84, 145.61, 148.86, 165.22, 177.18. HRMS (ESI^–^): calc. for C_18_H_20_BN_4_O_4_ [M – H]^−^: 367.1583, found: 367.1580.

### ((*R*)-Cyclopentyl­((*S*)-3-phenyl-2-(pyrazine-2-carboxamido)­propanamido)­methyl)­boronic Acid (**5l**)

Synthesized according to the General procedure from **3l** (17 mg, 0.1 mmol) to afford the title compound as a white solid (18 mg, 45% yield). The d.r. between (*R*,*S*) and (*S*,*S*) was 93:7. ^1^H NMR (400 MHz, MeOD) δ 0.95 – 1.12 (m, 2H, *c*Pe-CH_2_), 1.42 – 1.49 (m, 1H, *c*Pe-CH), 1.49 – 1.60 (m, 4H, *c*Pe-CH_2_), 1.73 – 1.85 (m, 2H, *c*Pe-CH_2_), 2.40 (d, *J* = 9.4 Hz, 1H, CH-B), 3.25 (d, *J* = 7.7 Hz, 2H, CH_2_-Ph), 5.05 (t, *J* = 7.6 Hz, 1H, CH-Bn), 7.21 – 7.26 (m, 1H, Ar-H), 7.27 – 7.33 (m, 4H, Ar-H), 8.70 (dd, *J* = 2.5, 1.5 Hz, 1H, Pyz-H), 8.80 (d, *J* = 2.5 Hz, 1H, Pyz-H), 9.18 (d, *J* = 1.5 Hz, 1H, Pyz-H). ^13^C NMR (101 MHz, MeOD) δ 26.02, 26.15, 31.49, 32.49, 38.80, 42.11, 52.92, 128.28, 129.75, 130.56, 137.06, 144.83, 144.87, 145.64, 148.91, 165.15, 176.86. HRMS (ESI^–^): calc. for C_17_H_20_BN_4_O_4_ [M – H]^−^: 395.1896, found: 395.1895.

### Residual Activity Measurements

The measurements of the residual activity of control compounds were carried out at final compound concentrations of 1 μM in the assay buffer (50 mM Tris-HCl, 0.5 mM ethylenediaminetetraacetic acid (EDTA), pH 7.4). Stock solutions of the compounds were prepared in DMSO. To the 35 μL of solution of the compound in the buffer, 35 μL of 0.4 nM human iCP (South Bay Bio, San Jose, CA, USA) or human cCP (Boston Biochem, Inc., Cambridge, MA, USA) was added. For assays evaluating the activity of β2c and β2i, the buffer was modified by adding the proteasomal activator PA28β (Boston Biochem, Inc., Cambridge, MA, USA) at a final concentration of 6 nM. After incubation at 37 °C for 30 min, the reaction was started by adding relevant fluorogenic substrates to final concentrations of 25 μM, which undergo preferential processing by appropriate immuno/proteasome subunits. The substrates used were acetyl-Nle-Pro-Nle-Asp-7-amino-4-methylcoumarin (Ac-nLPnLD-AMC, Bachem, Bubendorf, Switzerland) for β1c, acetyl-Pro-Ala-Leu-7-amino-4-methylcoumarin (Ac-PAL-AMC, Boston Biochem, Inc., Cambridge, MA, USA) for β1i, acetyl-Arg-Leu-Arg-7-amino-4-methylcoumarin (Ac-RLR-AMC, Bachem, Bubendorf, Switzerland) for β2c and β2i, acetyl-Trp-Leu-Ala-7-amino-4-methylcoumarin (Ac-WLA-AMC, Bachem, Bubendorf, Switzerland) for β5c, and acetyl-Ala-Asn-Trp-7-amino-4-methylcoumarin (Ac-ANW-AMC, Bachem, Bubendorf, Switzerland) for β5i. Progress of the reaction was measured using a BioTek Synergy HT microplate reader, monitoring fluorescence at 460 nm (excitation at 360 nm) for 60 min at 37 °C. The initial linear ranges were used to calculate the reaction velocity and determine the residual activity.

### Determination of IC_50_ Values

The final assay mixtures contained 0.4 nM human iCP or cCP in assay buffer (50 mM Tris-HCl, 0.5 mM EDTA, pH 7.4, addition of PA28β for β2c and β2i subunits). The solutions of the inhibitors were added to the black 96-well plates at different concentrations (DMSO concentration did not exceed 1%). After incubation at 37 °C for 30 min, the reaction was initiated by the addition of the substrate Ac-nLPnLD-AMC for β1c, Ac-PAL-AMC for β1i, Ac-RLR-AMC for β2c and β2i, Ac-WLA-AMC for β5c, and Ac-ANW-AMC for β5i. Fluorescence was monitored at 460 nm (excitation at 360 nm) for 60 min at 37 °C. The reaction progress was recorded and the initial linear phases were used to determine the velocity. The IC_50_ values were calculated using GraphPad Prism software (GraphPad Software, San Diego, CA, USA), and the results represent the mean values of at least three independent determinations.

### Cell-Based Proteasome-Glo Assay

The proteasome inhibitory activity of compound **5l** and bortezomib in intact cells was evaluated using the Proteasome-Glo Cell-Based Assay (Promega, Wisconsin, USA) according to the manufacturer’s instructions. Briefly, PBMCs were seeded into white, opaque 384-well plates (20 μL per well at density 5 × 10^3^) and treated with test compounds or vehicle (DMSO) for 6 h at 37 °C. After incubation, 25 μL of Proteasome-Glo reagent specific for the β5c and β5i subunits was added directly to each well. The plates were gently mixed and incubated for 10 min at room temperature, after which luminescence was measured using a plate luminometer (Tecan Spark microplate reader). Background signal from cell-free wells was subtracted, and the relative proteasome activity was normalized to the DMSO control (set to 100%). The IC_50_ values were determined from dose–response curves generated using nonlinear regression (four-parameter logistic fit) from three independent experiments.

### Cell Lines

All cell lines were obtained from ATCC (Manassas, Virginia, USA) and the German Collection of Microorganisms and Cell Cultures GmbH (DSMZ, Braunschweig, Germany), and maintained in RPMI-1640 or DMEM medium (Merck KGaA, Darmstadt, Germany) containing 10% fetal bovine serum (FBS) and supplemented with 1% penicillin/streptomycin and 1% l-glutamine. All cell lines were routinely tested for mycoplasma contamination and confirmed to be mycoplasma-free.

### Peripheral Blood Mononuclear Cells

Buffy coats from venous blood of anonymized healthy donors were purchased from Blood Transfusion Centre of Slovenia. PBMCs were isolated by density gradient centrifugation with Ficoll-Paque (Pharmacia, Sweden). The isolated cells were resuspended in RPMI 1640 medium (Sigma-Aldrich, St. Louis/MO, United States) supplemented with 10% heat-inactivated FBS (Gibco, ThermoFisher Scientific, Waltham/MA, United States), 2 mM l-glutamine (Sigma-Aldrich, St. Louis/MO, United States), 100 U/mL penicillin (Sigma-Aldrich, St. Louis/MO, United States), and 100 μg/mL streptomycin (Sigma-Aldrich, St. Louis/MO, United States).

### Immunoblotting

PBMCs were lysed in RIPA lysis buffer (50 mM Tris–HCl, pH 8.0, 150 mM NaCl, 1% NP-40, 0.5% Na-deoxycholate, 0.1% SDS, 1 mM EDTA, 1× Halt Phosphatase inhibitor cocktail and 1× Halt Protease inhibitor cocktail (Thermo Scientific)). Afterward, the lysates were sonicated and centrifuged at 15,000× g at 4 °C for 20 min. Protein concentrations in PBMC lysates were determined using the DC protein assay (Bio-Rad, Hercules, CA, USA). Equal amounts of protein (20 μg) were separated by SDS-PAGE on 12,5% acrylamide/bis-acrylamide gels at 80 V for 20 min, followed by 120 V for 60 min. Proteins were transferred to PVDF membranes using the iBlot3 Dry Blotting System (Thermo Fischer Scientific). Membranes were blocked with 5% BSA in 1 × TTBS for 1 h at room temperature and incubated overnight at 4 °C with primary antibodies against: β5i (1:1000; #13726), β5c (1:1000; #12919), and β1c (1:1000; #13267; all from Cell Signaling Technology), β1i (1:1000; #ab187645; all from Abcam), β2c (1:1000, #ab154745), and β2i (1:1000, #ab183506), and β-actin (1:5000; #A5316; SigmaPrestige). After washing, membranes were incubated with HRP-conjugated secondary antibodies (antirabbit IgG or antimouse IgG, 1:10,000; Cell Signaling Technology) for 1 h at room temperature. Detection was performed using the SuperSignal West Femto Maximum Sensitivity Substrate (Thermo Fisher Scientific) and imaged with the UVITEC Cambridge Imaging System (UVITEC, Cambridge, UK). Band intensities were quantified using NineAlliance software (UVITEC) and normalized to β-actin.

### Cytotoxicity Assay

Cytotoxicity was determined using the MTS cell proliferation assay (CellTiter 96 AQueous One Solution Cell Proliferation Assay, Promega, Wisconsin, USA), which measures mitochondrial metabolic activity as a surrogate for cell viability. The assay was performed according to the manufacturer protocol. The cytotoxicity was performed on HepG2, MCF-7, MDA-MB-231, MOLT-4, MM.1S, THP-1, RAJI cell lines and on HUVECs as well as PBMCs. The cells were seeded in 96-well plates (15.000–30.000 cells/well). After 24 h incubation at 5% CO_2_ and 37 °C, the cells were treated either with DMSO (negative control) or solutions of the tested compounds in DMSO. After 24 or 72 h, MTS reagent was added. Cells were incubated for additional 2 h - 4 h, followed by the measurement of absorbance at 490 nm on the Tecan Spark microplate reader.

### Stimulation of PBMCs and Cytokine Analysis

PBMCs (1.5 × 10^6^/mL) were plated in culture media in 96-well round-bottom plates. After a 60 min treatment with compounds at 37 °C and 5% CO_2_, cells were stimulated with LPS (*Escherichia coli*, O111:B4, 1 μg/mL, Sigma) for 24 h. Supernatants were then collected and analyzed for cytokine release by Human Inflammatory Cytokine Cytometric Bead Array (BD Biosciences, New Jersey, USA).

The methods related to the determination of kinetic solubility, metabolic stability in human liver microsomes, Caco-2 permeability, and parallel artificial membrane permeability studies were performed by Bienta, the Enamine biology services. The detailed descriptions of these methods are provided in the Supporting Information.

## Supplementary Material





## Notes

Supporting figures and tables and representative ^1^H, ^13^C NMR, and UHPLC spectra (PDF)
Molecular formula strings (CSV)

## Abbreviations

AMC: 7-amino-4-methylcoumarin
CP: core particle
cCP: constitutive proteasome
HMDSO: hexamethyldisiloxane
iCP: immunoproteasome
KAT: potassium acyltrifuoroborate
LPS: lipopolysaccharide
PBMC: peripheral blood mononuclear cell
SAR: structure-activity relationship
TIM: trifluoroborate iminium
TAM: trifluoborate ammonium
TNF-α: tumor necrosis factor-α
