# Supplementary material for: α‑Aminoboronic Acid Moieties in Boro Dipeptides Modulate Proteasome Subunit Selectivity and Provide Access to Compounds with Potent Anticancer and Anti-Inflammatory Activity
Source: J Med Chem. 2025 Dec 4;68(24):26405–17. doi: 10.1021/acs.jmedchem.5c02548 (PMC12751025; doi:10.1021/acs.jmedchem.5c02548)
Supplement: Supplementary file 1 [file jm5c02548_si_001.pdf]

Supporting Information

α-Aminoboronic acid moieties in boro dipeptides modulate proteasome subunit selectivity and provide access to compounds with potent anti-cancer and anti-inflammatory activity

Nika Strašek Benedik,[a] Andrej Šterman,[a] Lara Smrdel,[a] Stane Pajk,[a] Stanislav Gobec,[a] Zdenko Časar,[a,b] Martina Gobec,[a,*] and Izidor Sosič[a,*]

[a] University of Ljubljana, Faculty of Pharmacy, Aškerčeva cesta 7, SI-1000 Ljubljana, Slovenia

[b] Lek Pharmaceuticals d.d., Sandoz Development Center Slovenia, Verovškova ulica 57, 1526 Ljubljana, Slovenia

* Corresponding authors

Martina Gobec: martina.gobec@ffa.uni-lj.si

Izidor Sosič: izidor.sosic@ffa.uni-lj.si.

Contents

1. Supporting Figures S2

2. Supporting Tables S10

3. Methods...S13

4. Representative NMR, MS, and UHPLC Spectra S15

5. References S25

1. Supporting Figures

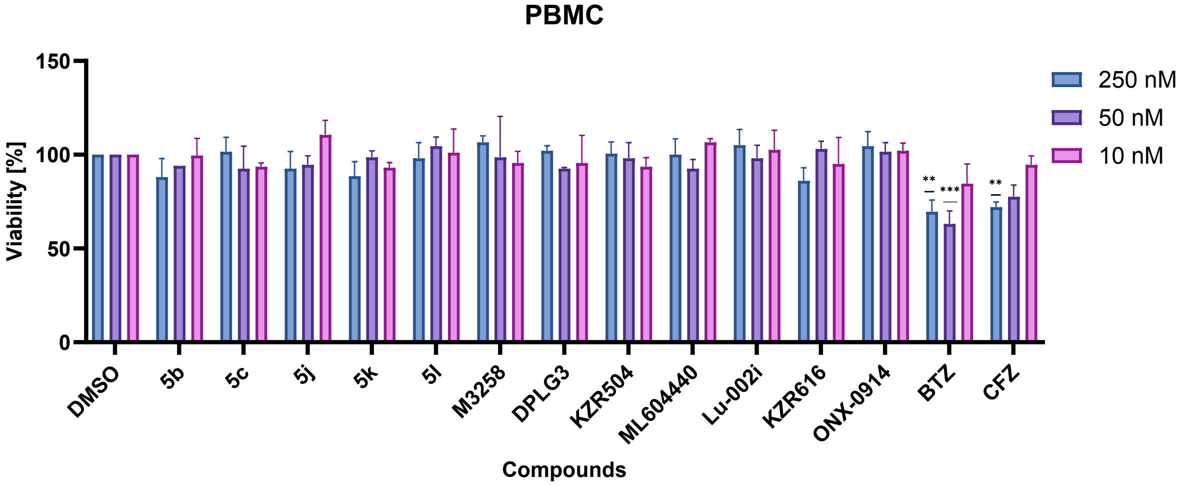

Figure S1. The cytotoxic effect of analogs **5b**, **5c**, **5j–l**, subunit-selective control compounds, bortezomib, and carfilzomib on PBMCs. The compounds were tested at 10 nM, 50 nM, and 250 nM. The cells were treated with compounds at indicated concentrations for 24 h, and cytotoxicity was assessed by MTS assay, which measures mitochondrial metabolic activity as a surrogate for cell viability. Data were normalized to DMSO controls and are presented as means ± SD of five individual PBMC donors (N = 5). Statistical significance relative to DMSO controls was calculated using two-way ANOVA post hoc Dunnett's test. A p-value of less than 0.05 was considered significant (***p < 0.001; **p < 0.01; *p < 0.05).

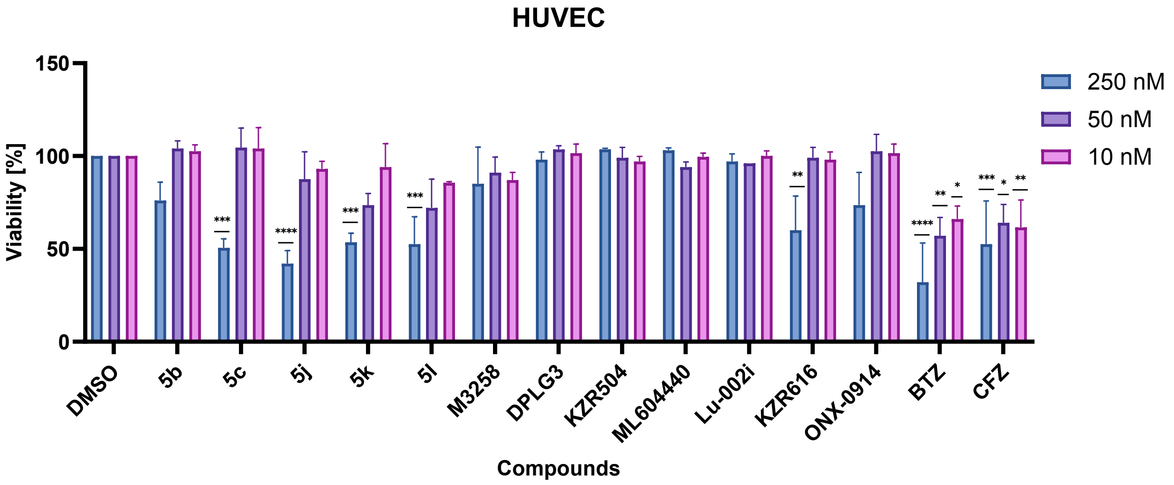

Figure S2. The cytotoxic effect of analogs **5b**, **5c**, **5j–l**, subunit-selective control compounds, bortezomib, and carfilzomib on HUVECs. The compounds were tested at 10 nM, 50 nM, and 250 nM. The cells were treated with compounds at indicated concentrations for 72 h, and cytotoxicity was assessed by MTS assay, which measures mitochondrial metabolic activity as a surrogate for cell viability. Data were normalized to DMSO controls and are presented as means ± SD of three independent experiments (N = 3). Statistical significance relative to DMSO controls was calculated using two-way ANOVA post hoc Dunnett's test. A p-value of less than 0.05 was considered significant (****$p < 0.0001$; ***$p < 0.001$; **$p < 0.01$; *$p < 0.05$).

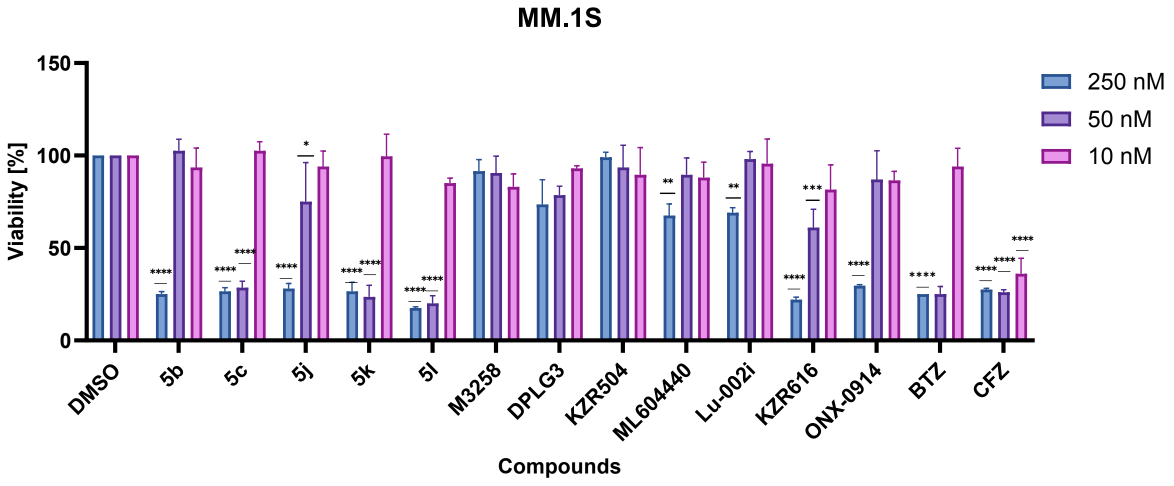

Figure S3. The cytotoxic effect of analogs **5b**, **5c**, **5j–l**, subunit-selective control compounds, bortezomib, and carfilzomib on MM.1S. The compounds were tested at 10 nM, 50 nM, and 250 nM. The cells were treated with compounds at indicated concentrations for 72 h, and cytotoxicity was assessed by MTS assay, which measures mitochondrial metabolic activity as a surrogate for cell viability. Data were normalized to DMSO controls and are presented as means ± SD of three

independent experiments (N = 3). Statistical significance relative to DMSO controls was calculated using two-way ANOVA post hoc Dunnett's test. A p-value of less than 0.05 was considered significant (****p < 0.0001; ***p < 0.001; **p < 0.01; *p < 0.05).

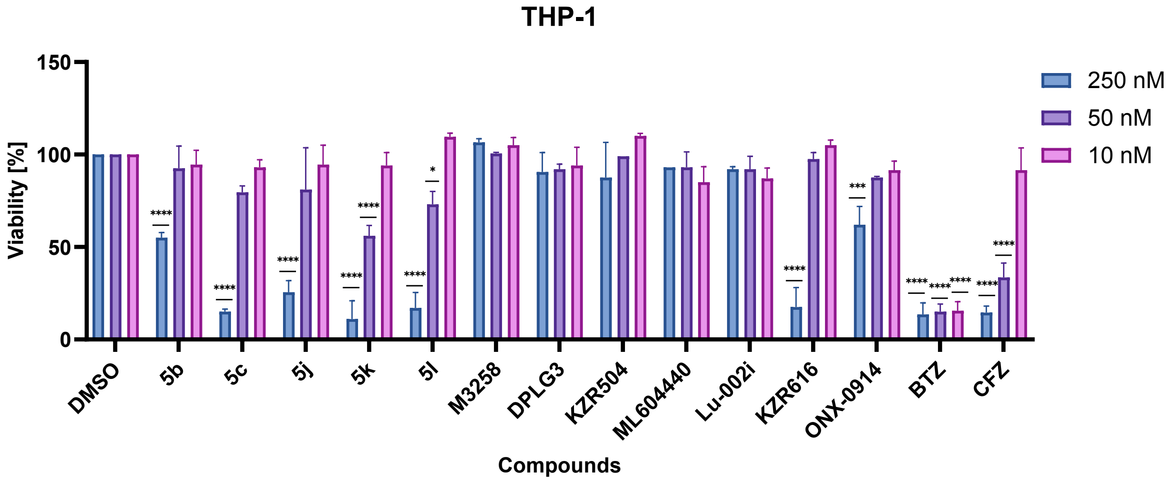

Figure S4. The cytotoxic effect of analogs **5b**, **5c**, **5j–l**, subunit-selective control compounds, bortezomib, and carfilzomib on THP-1. The compounds were tested at 10 nM, 50 nM, and 250 nM. The cells were treated with compounds at indicated concentrations for 72 h, and cytotoxicity was assessed by MTS assay, which measures mitochondrial metabolic activity as a surrogate for cell viability. Data were normalized to DMSO controls and are presented as means ± SD of three independent experiments (N = 3). Statistical significance relative to DMSO controls was calculated using two-way ANOVA post hoc Dunnett's test. A p-value of less than 0.05 was considered significant (****p < 0.0001; ***p < 0.001; **p < 0.01; *p < 0.05).

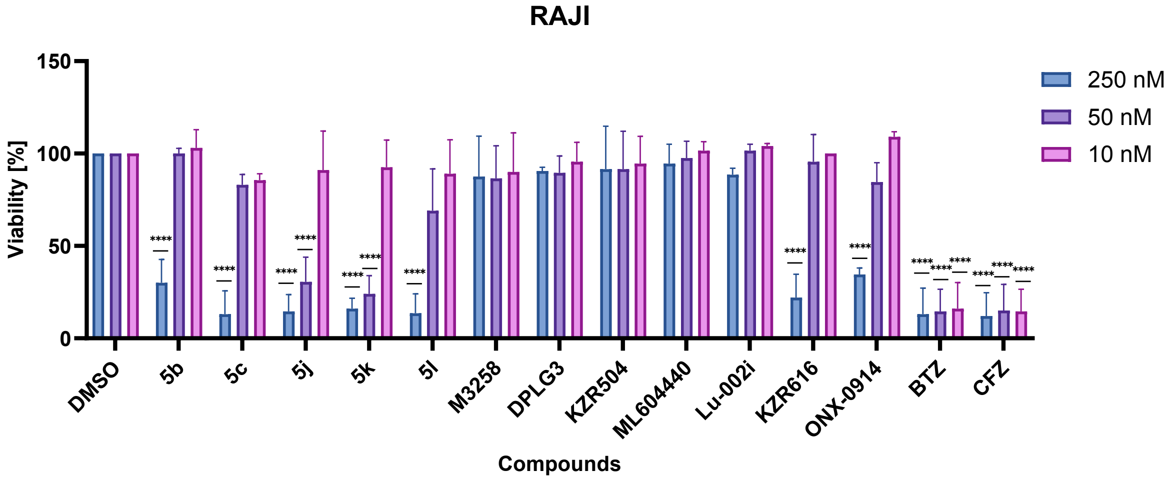

Figure S5. The cytotoxic effect of analogs **5b**, **5c**, **5j–l**, subunit-selective control compounds,

bortezomib, and carfilzomib on RAJI. The compounds were tested at 10 nM, 50 nM, and 250 nM. The cells were treated with compounds at indicated concentrations for 72 h, and cytotoxicity was assessed by MTS assay, which measures mitochondrial metabolic activity as a surrogate for cell viability. Data were normalized to DMSO controls and are presented as means ± SD of three independent experiments (N = 3). Statistical significance relative to DMSO controls was calculated using two-way ANOVA post hoc Dunnett's test. A p-value of less than 0.05 was considered significant (****p < 0.0001; ***p < 0.001; **p < 0.01; *p < 0.05).

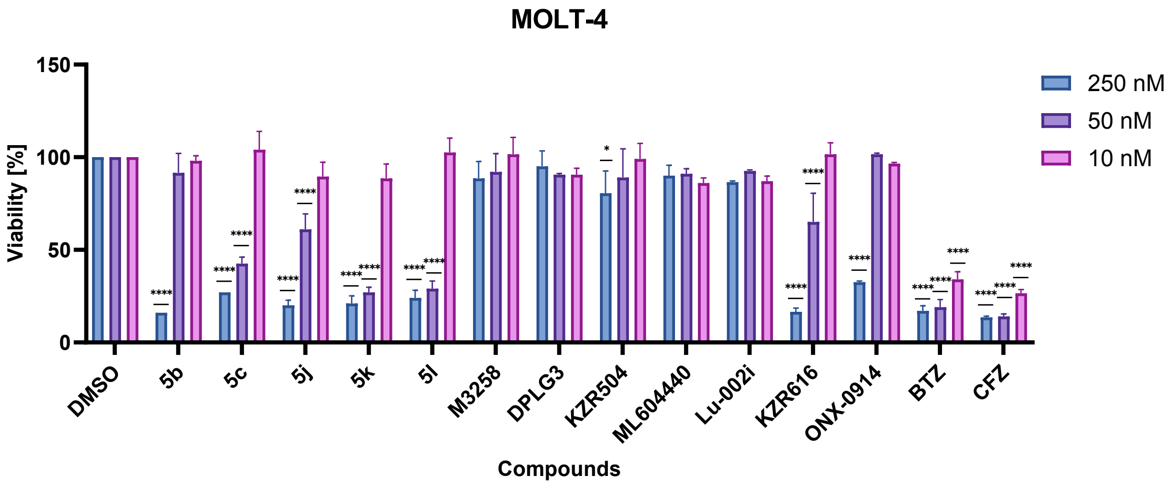

Figure S6. The cytotoxic effect of analogs **5b**, **5c**, **5j–l**, subunit-selective control compounds, bortezomib, and carfilzomib on MOLT-4. The compounds were tested at 10 nM, 50 nM, and 250 nM. The cells were treated with compounds at indicated concentrations for 72 h, and cytotoxicity was assessed by MTS assay, which measures mitochondrial metabolic activity as a surrogate for cell viability. Data were normalized to DMSO controls and are presented as means ± SD of three independent experiments (N = 3). Statistical significance relative to DMSO controls was calculated using two-way ANOVA post hoc Dunnett's test. A p-value of less than 0.05 was considered significant (****p < 0.0001; ***p < 0.001; **p < 0.01; *p < 0.05).

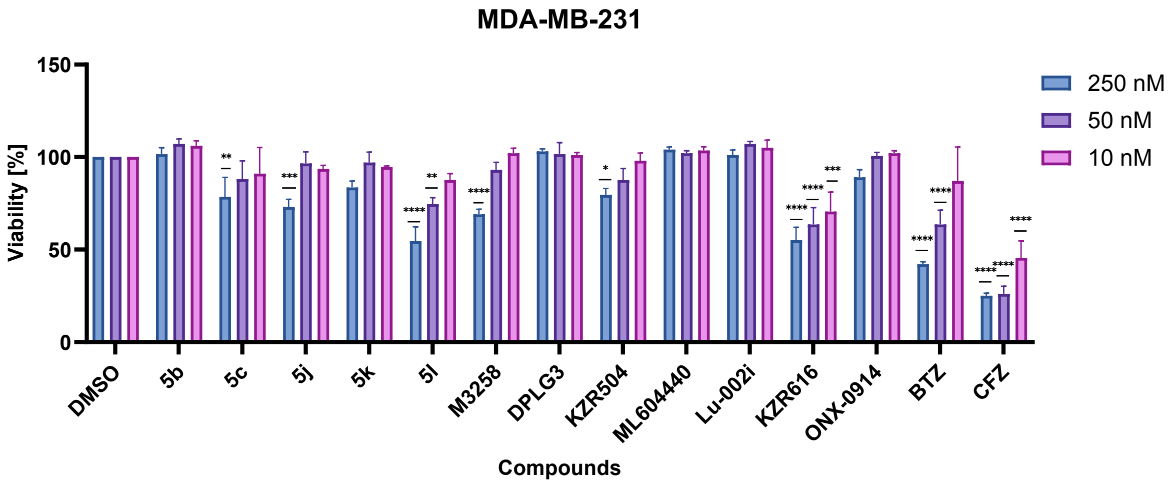

Figure S7. The cytotoxic effect of analogs **5b**, **5c**, **5j–l**, subunit-selective control compounds, bortezomib, and carfilzomib on MDA-MB-231. The compounds were tested at 10 nM, 50 nM, and 250 nM. The cells were treated with compounds at indicated concentrations for 72 h, and cytotoxicity was assessed by MTS assay, which measures mitochondrial metabolic activity as a surrogate for cell viability. Data were normalized to DMSO controls and are presented as means ± SD of three independent experiments (N = 3). Statistical significance relative to DMSO controls was calculated using two-way ANOVA post hoc Dunnett's test. A p-value of less than 0.05 was considered significant (****$p < 0.0001$; ***$p < 0.001$; **$p < 0.01$; *$p < 0.05$).

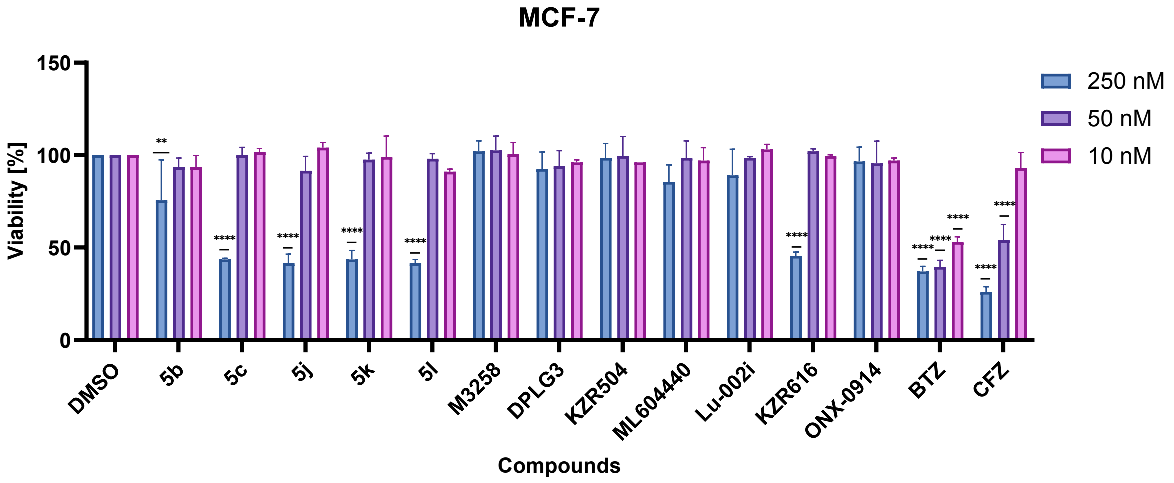

Figure S8. The cytotoxic effect of analogs **5b**, **5c**, **5j–l**, subunit-selective control compounds, bortezomib, and carfilzomib on MCF-7. The compounds were tested at 10 nM, 50 nM, and 250 nM. The cells were treated with compounds at indicated concentrations for 72 h, and cytotoxicity was assessed by MTS assay, which measures mitochondrial metabolic activity as a surrogate for cell viability. Data were normalized to DMSO controls and are presented as means ± SD of three

independent experiments (N = 3). Statistical significance relative to DMSO controls was calculated using two-way ANOVA post hoc Dunnett's test. A p-value of less than 0.05 was considered significant (****p < 0.0001; ***p < 0.001; **p < 0.01; *p < 0.05).

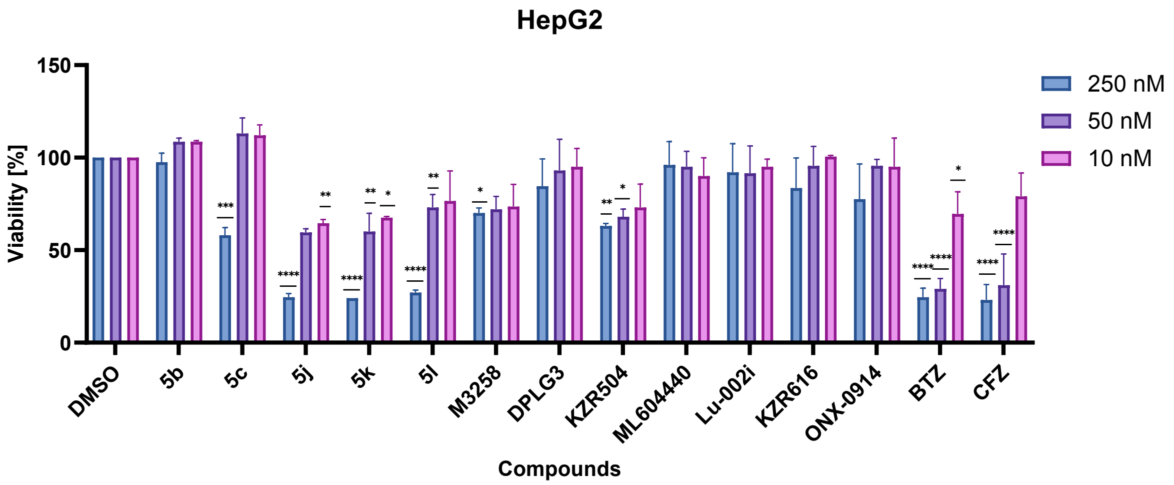

Figure S9. The cytotoxic effect of analogs **5b**, **5c**, **5j–l**, subunit-selective control compounds, bortezomib, and carfilzomib on HepG2. The compounds were tested at 10 nM, 50 nM, and 250 nM. The cells were treated with compounds at indicated concentrations for 72 h, and cytotoxicity was assessed by MTS assay, which measures mitochondrial metabolic activity as a surrogate for cell viability. Data were normalized to DMSO controls and are presented as means ± SD of three independent experiments (N = 3). Statistical significance relative to DMSO controls was calculated using two-way ANOVA post hoc Dunnett's test. A p-value of less than 0.05 was considered significant (****p < 0.0001; ***p < 0.001; **p < 0.01; *p < 0.05).

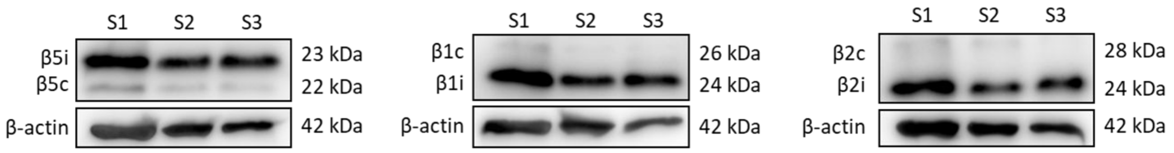

Figure S10. Protein levels of catalytically active constitutive proteasome and immunoproteasome subunits in PBMCs from three healthy donors (N = 3). β-actin was used as the loading control.

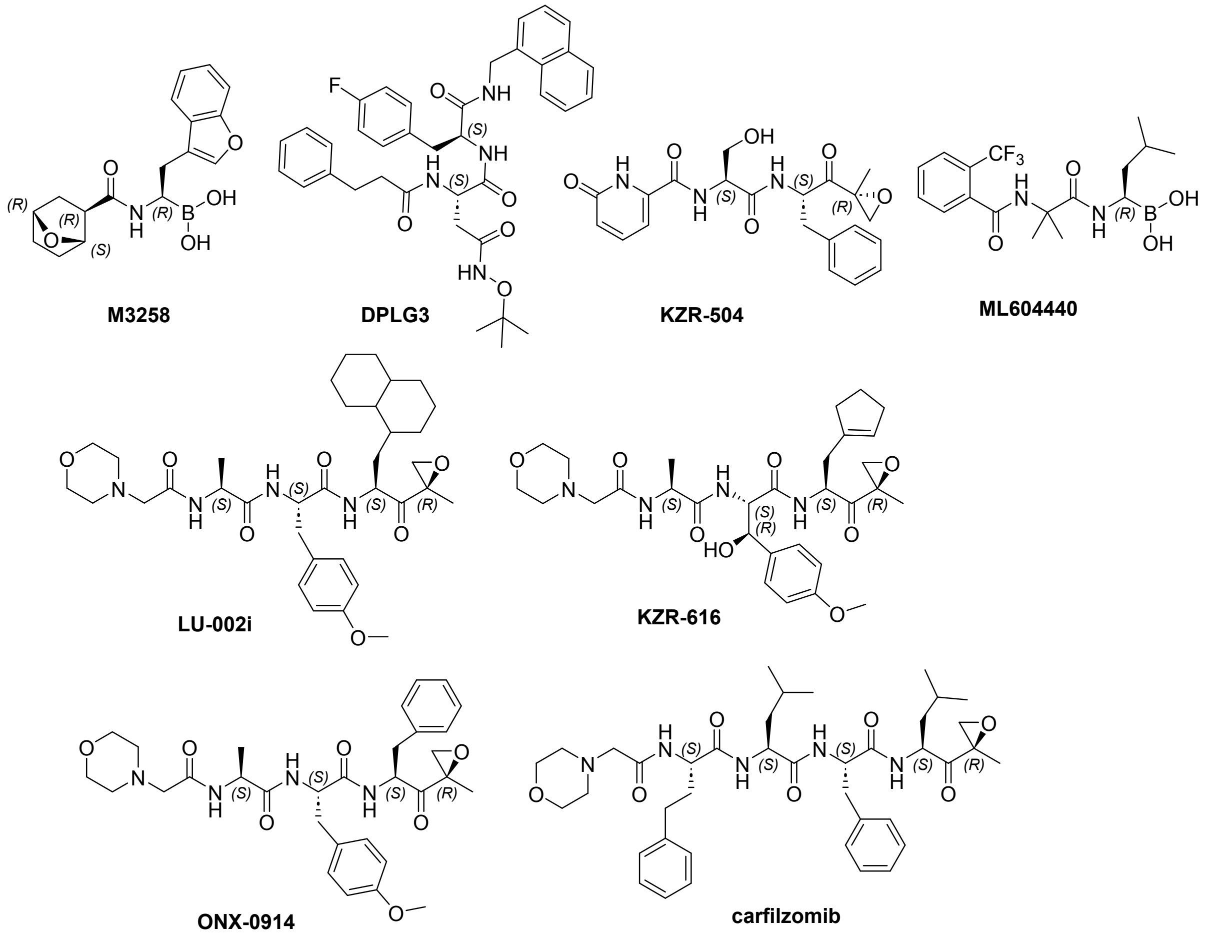

Figure S11. Structures of control compounds used in the study. β1i-selective (KZR-504, ML604440), β2i-selective (Lu-002i), β5i-selective (M3258, DPLG3), β1i/β5i-targeting (KZR-616), β5c/β5i-targeting (ONX-0914), and pan-inhibitor (carfilzomib).

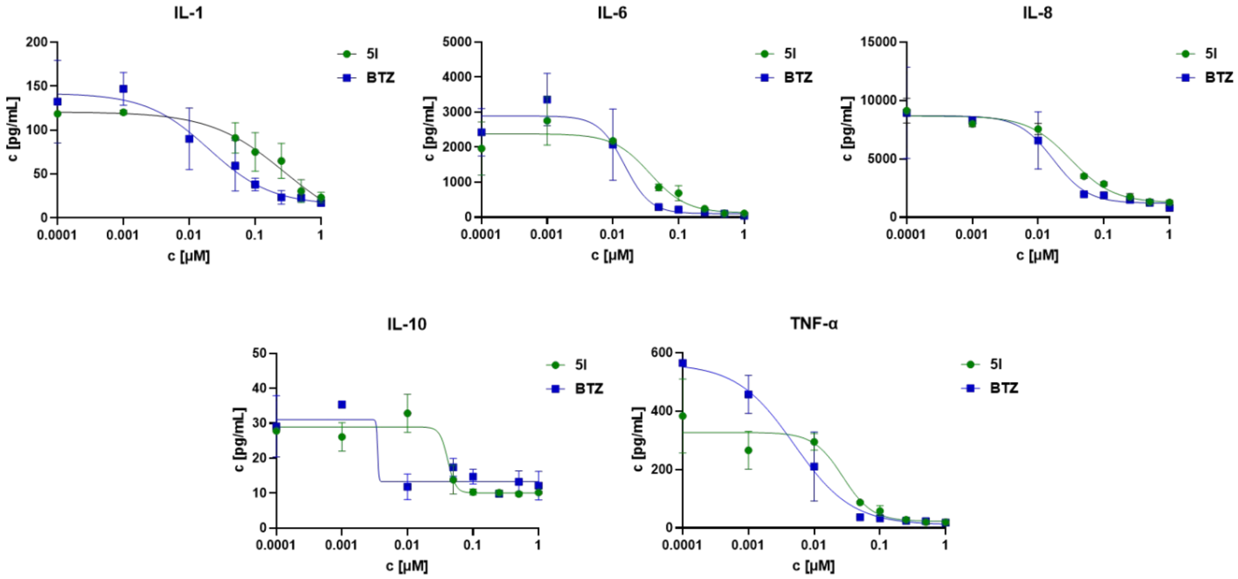

Figure S12. Dose-dependent effects on inflammatory cytokine release in LPS-stimulated PBMCs for compounds **5l** and bortezomib. The cells were pre-treated for 1 h with inhibitors, followed by the addition of LPS (1 μg/mL). As the negative control (designated by 'DMSO' in Figure legends), cells were treated only with DMSO, followed by the addition of LPS (1 μg/mL). The concentrations of cytokines were determined in the supernatants after additional 24 h treatment. The results are represented as means ± SD of three independent experiments (N = 3).

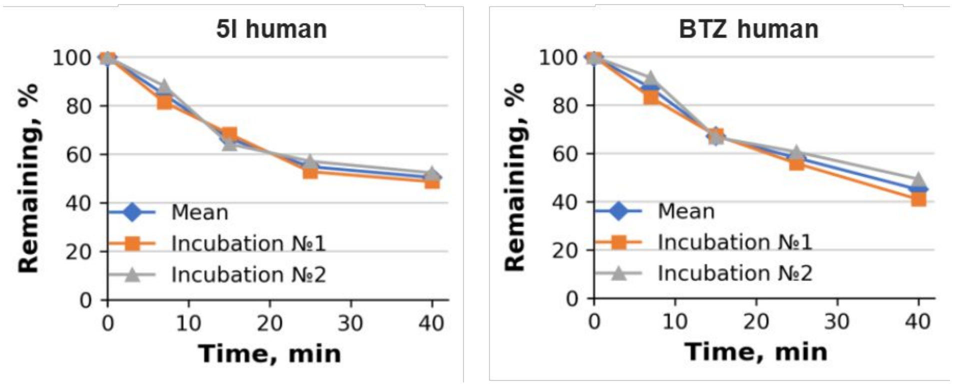

Figure S13. Human microsomal stability for **5l** and bortezomib (BTZ). The following values were obtained for **5l**: k_{el} = 0.018 min^{-1}, $t_{1/2}$ = 39.4 min, Cl_{int} = 42 μL/min/mg. The following values were obtained for bortezomib: k_{el} = 0.020 min^{-1}, $t_{1/2}$ = 34.5 min, Cl_{int} = 48 μL/min/mg.

2. Supporting Tables

Table S1. *In vitro* inhibitory potencies (IC_{50} values ± SD) of compounds against all subunits of the human cCP and iCP. For comparison, the IC_{50} values of bortezomib are added.

Compound	β1c	β1i	β2c	β2i	β5c	β5i
**5a**	NA	0,746 ± 0,315	NA	NA	12,8 ± 6,1	0,22 ± 0,061
**5b**	0,545 ± 0,105	0,042 ± 0,021	NA	9,8 ± 2,2	0,712 ± 0,551	0,032 ± 0,024
**5c**	0,526 ± 0,427	0,038 ± 0,018	NA	NA	0,225 ± 0,154	0,032 ± 0,01
**5d**	0,897 ± 0,323	0,017 ± 0,013	1,847 ± 0,199	74,5 ± 10	0,114 ± 0,044	0,003 ± 0,01
**5e**	1,337 ± 0,7	0,040 ± 0,013	79 ± 4	10,4 ± 1	0,402 ± 0,294	0,015 ± 0,002
**5f**	NA	22,4 ± 9,8	NA	NA	3,3 ± 4	1,3 ± 1,1
**5g**	0,315 ± 0,019	0,201 ± 0,064	45 ± 22,8	8,6 ± 0,4	0,183 ± 0,205	0,018 ± 0,003
**5h**	1,308 ± 0,247	0,27 ± 0,086	NA	0,26 ± 0,292	0,012 ± 0,007	0,024 ± 0,017
**5i**	0,235 ± 0,074	0,054 ± 0,043	4,9 ± 2,2	3,4 ± 2,6	0,054 ± 0,041	0,017 ± 0,012
**5j**	0,021 ± 0,006	0,002 ± 0,001	3,4 ± 0,1	1,278 ± 0,098	0,011 ± 0,005	0,019 ± 0,034
**5k**	0,827 ± 0,234	0,172 ± 0,057	0,44 ± 0,172	0,499 ± 0,005	0,005 ± 0,002	0,009 ± 0,007
**5l**	0,018 ± 0,013	0,003 ± 0,002	37,4 ± 4,3	1 ± 0,05	0,044 ± 0,037	0,058 ± 0,048
**bortezomib**	0,010 ± 0,003	0,004 ± 0,001	1,3 ± 0,3	0,49 ± 0,022	0,002 ± 0,001	0,001 ± 0,001

NA, not active

Table S2. IC_{50} values (μM) of control compounds used in the study. Because IC_{50} values depend on type of assay used and specific assay conditions (enzyme concentration, pre-incubation time, substrate used), we provide the IC_{50} values from our own assay. Previously published data extracted from ref. [1] (in parentheses, IC_{50} in μM) is included as well. Literature data for carfilzomib and bortezomib are from ref. [2].

Cpd	β1c	β1i	β2c	β2i	β5c	β5i
**M3258**<br>β5i-selective	> 1<br>(> 30)	> 1<br>(> 30)	> 1<br>(> 30)	> 1<br>(> 30)	1.229 ± 0.09<br>(2.519)	0.0074 ± 0.001<br>(0.0041)
**DPLG3**<br>β5i-selective	> 1<br>(> 33.3)	> 1<br>(> 33.3)	> 1<br>(> 33.3)	> 1<br>(> 33.3)	> 1<br>(32.4)	0.011 ± 0.002<br>(0.0045)
**KZR-504**<br>β1i-selective	> 1<br>(46.35)	0.059 ± 0.011<br>(0.051)	> 1<br>(> 250)	> 1<br>(> 250)	> 1<br>(6.9)	> 1<br>(4.3)
**ML604440**<br>β1i-selective	> 1<br>(n.r.)	0.008 ± 0.005<br>(~0.0125)	> 1<br>(n.r.)	> 1<br>(n.r.)	> 1<br>(> 1)	> 1<br>(> 1)
**Lu-002i**<br>β2i-selective	n.d.<br>(> 100)	n.d.<br>(> 100)	n.d.<br>(12.1)	n.d.<br>(0.18)	n.d.<br>(> 100)	n.d.<br>(> 100)
**KZR-616**<br>β5i/β1i-selective	> 1<br>(> 10.6)	0.096 ± 0.032<br>(0.131)	0.403 ± 0.151<br>(0.604)	0.338 ± 0.122<br>(0.623)	0.426 ± 0.048<br>(0.688)	0.026 ± 0.021<br>(0.039)
**ONX-0914**<br>β5c/β5i-selective	> 1<br>(> 10)	0.336 ± 0.194<br>(0.46)	> 1<br>(1.1)	0.852 ± 0.058<br>(0.59)	0.082 ± 0.025<br>(0.054)	0.022 ± 0.007<br>(0.0057)
**carfilzomib**<br>pan	0.038 ± 0.005<br>(0.013)	0.111 ± 0.060<br>(0.069)	0.098 ± 0.021<br>(0.043)	0.078 ± 0.038<br>(0.013)	0.002 ± 0.001<br>(0.0021)	0.004 ± 0.002<br>(0.0032)
**bortezomib**<br>pan	0.010 ± 0.003<br>(0.025)	0.004 ± 0.001<br>(0.0029)	1.3 ± 0.3<br>(12.77)	0.49 ± 0.022<br>(1.63)	0.002 ± 0.001<br>(0.025)	0.001 ± 0.001<br>(0.0029)

n.d. Not determined

Table S3. The kinetic solubility data of **5l** and bortezomib

**Compound**	**PBS solubility, pH 7.4, μM**
**5l**	≥ 200
bortezomib	399

Table S4. A-B and B-A permeability data as well as the efflux ratio (PappB-A/PappA-B) data for **5l** and bortezomib.

**Compound**	**Papp (AB), 10^{-6} cm/s**	**Papp (BA), 10^{-6} cm/s**	**Efflux ratio***
**5l**	2.0 ± 0.2	17.1 ± 1	8.4
bortezomib	2.8 ± 0.1	20.7 ± 0.1	7.3

* Efflux ratio is expressed as the quotient of Papp(BA) to Papp(AB)

Table S5. Gastrointestinal tract parallel artificial membrane permeability assay (PAMPA) for **5l** and bortezomib.

**Compound**	**Permeability, Log_{10} [cm/s]**
**5l**	-4.8 ± 0.06*
bortezomib	-4.8 ± 0.02*

P_e = 15.8 × 10^6 cm/s

Table S6. Plasma protein binding data for **5l** and bortezomib.

**Compound**	**% of bound compound**	Recovery [%]	Stability [%]
**5l**	91	93	86
bortezomib	93	100	92

3. Methods

Kinetic solubility

Briefly, using a 20 mM (10 mM for **5l**) stock solution of the compound in 100% DMSO, dilutions were prepared to a theoretical concentration of 400 µM (200 µM for **5l**) in duplicates in phosphate-buffered saline pH 7.4 (138 mM NaCl, 2.7 mM KCl, 10 mM K-phosphate) with 2% final DMSO. The experimental compound dilutions in PBS were further allowed to equilibrate at 25 °C on a thermostatic shaker for two hours and then filtered through HTS filter plates using a vacuum manifold. The filtrates of test compounds were diluted 2-fold with acetonitrile with 2% DMSO before measuring.

In parallel, using a 20 mM (10 mM for **5l**) stock solution of the compound in 100% DMSO dilutions were prepared to theoretical concentrations of 0 µM (blank), 10 µM, 25 µM, 50 µM, 100 µM, and 200 µM (0 µM (blank), 10 µM, 25 µM, 50 µM, and 100 µM for **5l**) in 50% acetonitrile/PBS with 2% final DMSO to generate calibration curves. Ondansetron was used as a reference compound to control proper assay performance. 200 µL of each sample was transferred to a 96-well plate and measured in the 230-550 nm range with a 5 nm step.

Metabolic stability in human liver microsomes

Microsomal incubations were carried out in 96-well plates in 5 aliquots of 30 µL each (one for each time point). Liver microsomal incubation medium comprised of phosphate buffer (100 mM, pH 7.4), $MgCl_2$ (3.3 mM), NADPH (3 mM), glucose-6-phosphate (5.3 mM), glucose-6-phosphate dehydrogenase (0.67 units/ml) with 0.42 mg of liver microsomal protein per mL. In the control reactions, the NADPH-cofactor system was substituted with phosphate buffer. Test compounds (2 µM, final acetonitrile concentration 1.6 %) were incubated with microsomes at 37 °C, shaking at 100 rpm. Five time points over 40 min were analyzed. The reactions were stopped by adding 4 volumes of acetonitrile with internal standard to incubation aliquots, followed by protein sedimentation by centrifuging at 5500 rpm for 5 min. Each reaction was performed in duplicates. Supernatants were analyzed using the HPLC system coupled with a tandem mass spectrometer. The elimination constant (k_{el}), half-life ($t_{1/2}$), and intrinsic clearance (Cl_{int}) were determined in a plot of ln(AUC) versus time, using linear regression analysis.

Caco-2 permeability

Caco-2 cells were cultured in 75 cm^2 flasks to 80-90% confluence in a humidified atmosphere at 37 °C and 5% CO_2. Cells were detached with Trypsin/EDTA solution and resuspended in the complete medium containing DMEM high glucose (4500 mg/L) with L-glutamine (4 mM) supplemented with 10%

heat-inactivated Fetal Bovine Serum, 1% non-essential amino acids, and 730 nM puromycin and seeded at a density 5×10^5 cells in 75 cm^2 flask. After 5 days, cells were trypsinized and resuspended in the complete medium to a final concentration of 600×10^3 cells/mL. 400 µL of the cell suspension was added to each well of the HTS 24-Multiwell Insert System and 25 mL of prewarmed complete medium was added to the feeder tray. Caco-2 cells were incubated in Multiwell Insert System for 6-10 days before the transport experiments. The medium in the filter plate and feeder tray was refreshed every other day. The 24-well insert plate was removed from its feeder plate and placed in a new sterile 24-well transport analysis plate. The inserts were washed with PBS after medium aspiration. Ketoprofen, atenolol, and digoxin were used as reference compounds.

To determine the rate of compounds transport in apical (A)-to-basolateral (B) direction, 300 µL of the test compound dissolved in transport buffer (Hanks' BSS (9.5 g/L) and $NaHCO_3$ (0.35 g/L) with $MgSO_4$ to final concentration 0.81 mM, $CaCl_2$ to final concentration 1.26 mM, HEPES to final concentration 25 mM, pH adjusted to 7.4) was added into the filter wells; 1000 µL of transport buffer was added to transport analysis plate wells. To determine transport rates in the basolateral (B)-to-apical (A) direction, 1000 µL of the test compound solutions was added into the wells of the transport analysis plate, the wells in the filter plate were filled with 300 µL of buffer. The final amount of reference and test compounds was 10 µM. The plates were incubated for 90 min at 37 °C under continuous shaking at 100 rpm. 75 µL aliquots were taken from the donor and receiver compartments for LC-MS/MS analysis. All samples were mixed with 2 volumes of acetonitrile followed by protein sedimentation by centrifuging at 10000 rpm for 10 min. Supernatants were analyzed using the HPLC system coupled with a tandem mass spectrometer.

Gastrointestinal tract parallel artificial membrane permeability (PAMPA) assay

All steps of the PAMPA were carried out according to the pION Inc. PAMPA Explorer™ Manual. The main principle of the assay is the incubation of the compound in the donor chamber (a well in the Donor Plate) with an aqueous buffer, which is separated from the acceptor chamber (a well in the Acceptor Plate) with another buffer by a phospholipid or hydrocarbon membrane fixed on a filter support. After the test, concentrations in the corresponding donor and acceptor wells are measured, and permeability is calculated. The GIT model was simulated using the GIT-0 phospholipid mix. Verapamil, quinidine (high permeability), and ranitidine (low permeability) were used as reference compounds.

All compounds were tested in triplicate. Prisma HT buffer (pH 7.4) containing 50 µM test compounds (0.5% DMSO) was added to the Donor Plate wells. Acceptor Sink Buffer was added to each well of the Acceptor Plate. Incubation was done at room temperature for 4 hours without stirring. After

incubation, aliquots from both plates were transferred to optic UV-Vis plates, and optic plates were read on a microplate reader in absorbance mode in the range of 230-550 nm with a 4 nm step. Then, the apparent permeability coefficient was calculated.

Human plasma protein binding

The assay was performed in a multiple-use 96-well dialysis unit (HTD96b dialyzer). Each individual well unit consisted of two chambers separated by a vertically aligned dialysis membrane of predetermined pore size (MWCO 14 kDa). 125 µL of non-diluted plasma spiked with the compound (1 µM, final DMSO and acetonitrile concentration was 0.005% and 1%, respectively) was added to one chamber and the same volume of PBS buffer pH 7.4 to the other chamber. HTD96b dialyzer was incubated with shaking (250 rpm) at 37 °C, 5% CO_2, and a saturating humidity (~95%) for 5 h. For samples preparation, an aliquot of the content of each chamber had been mixed with the same volume of the blank opposite matrix. In order to define non-specific loss of the compound during this assay, standard solution was created by mixing an aliquot of spiked plasma with blank buffer without dialysis. Two aliquots of the standard solution were incubated with shaking (250 rpm) at 37 °C, 5% CO_2, and a saturating humidity (~95%) for 5 h (recovery samples). The other two aliquots were immediately diluted with acetonitrile and stored at 4 °C until LC-MS/MS analysis (stability samples). All samples were diluted 5-fold with 90% acetonitrile with internal standard with subsequent plasma proteins sedimentation by centrifuging at 6000 rpm for 5 min. Supernatants were analyzed using HPLC system coupled with tandem mass spectrometer. Verapamil was used as a reference compound to control proper assay performance.

4. Representative NMR, MS, and UHPLC Spectra

Bortezomib and its analogs are known to exist as the free boronic acid as well as dimeric and trimeric boronic anhydrides. These forms interconvert rapidly in solution and are indistinguishable by routine NMR experiments.[3] To the best of our knowledge, this equilibrium does not affect the biological activity of such compounds. On UHPLC, the interconverting boronic species were not chromatographically resolved; however, diastereomers were at least partially separated, yielding two distinct chromatographic signals with identical mass spectra. By contrast, the bortezomib reference standard produced a single chromatographic signal, consistent with its pure enantiomeric form (see **Supporting Figure 1**).

During LC-MS analysis, additional species were detected, likely arising from the energetic conditions of the ion source. In positive-ion mode, we observed signals attributable to oxaborane species, a cyclic B_2O_2 dimer (1,3-dioxa-2,4-diboretane), and boroxines.[4,5] The first two are intrinsically unstable and short-lived; however, related derivatives have been observed or isolated, and the energetic conditions of the ion source together with the MS vacuum enable detection of these otherwise transient species. In negative-ion mode, a formate adduct was observed, as expected due to the presence of formic acid in the mobile phase, along with a dimeric boronate species.[6] Representative chromatograms and mass spectra for bortezomib and compound **5l** are shown below in the **Supporting Figures 1 and 2**, respectively.

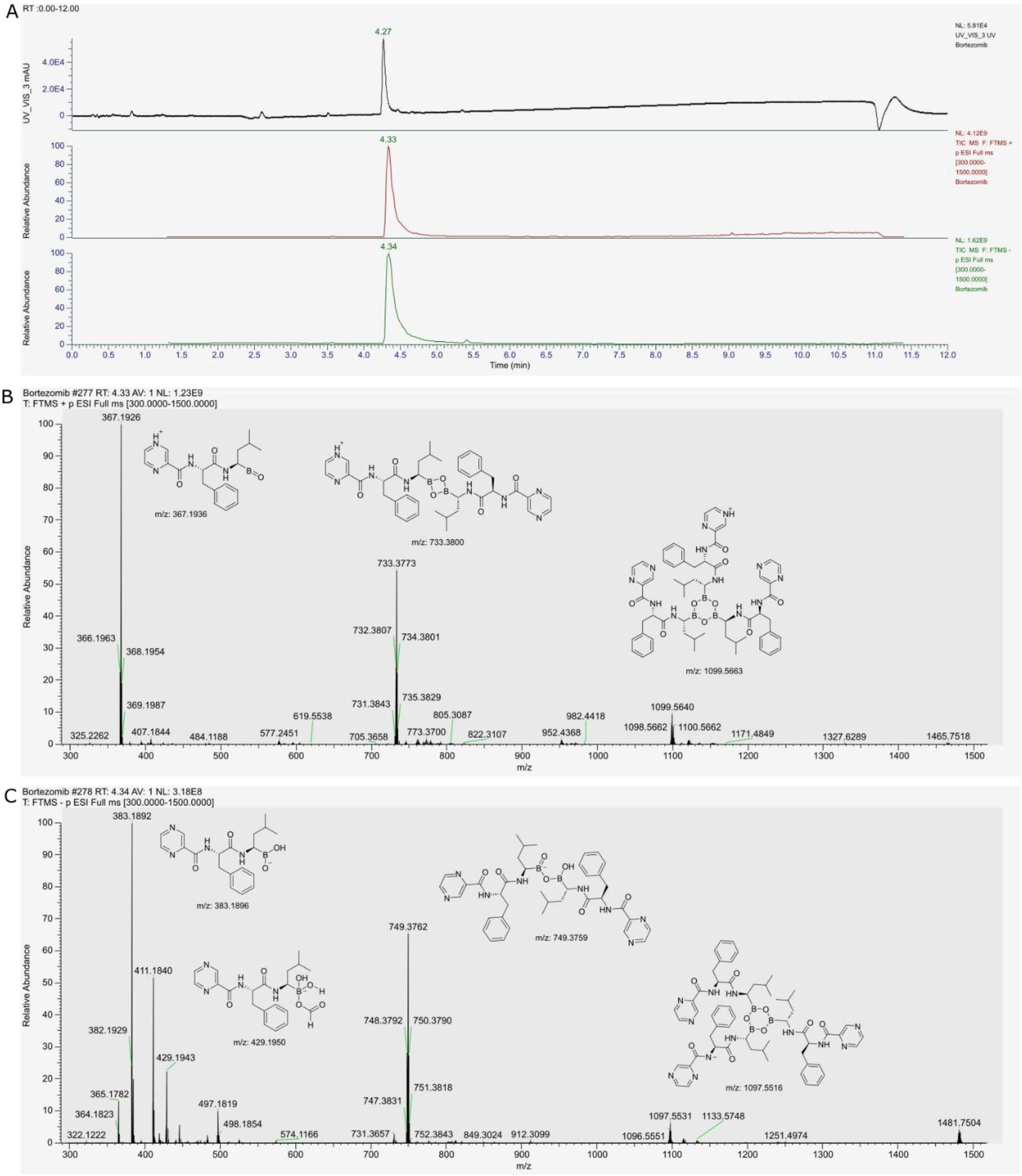

Supporting Figure 1. (A) From top to bottom: chromatogram of bortezomib recorded at 255 nm, total ion chromatogram (TIC) recorded in positive mode and TIC recorded in negative mode. (B) Positive-ion mass spectrum recorded at 4.33 min. (C) Negative-ion mass spectrum recorded at 4.34 min. Both mass spectra are annotated with the proposed structures of ions derived from bortezomib, showing calculated monoisotopic m/z values for the most abundant isotopologues.

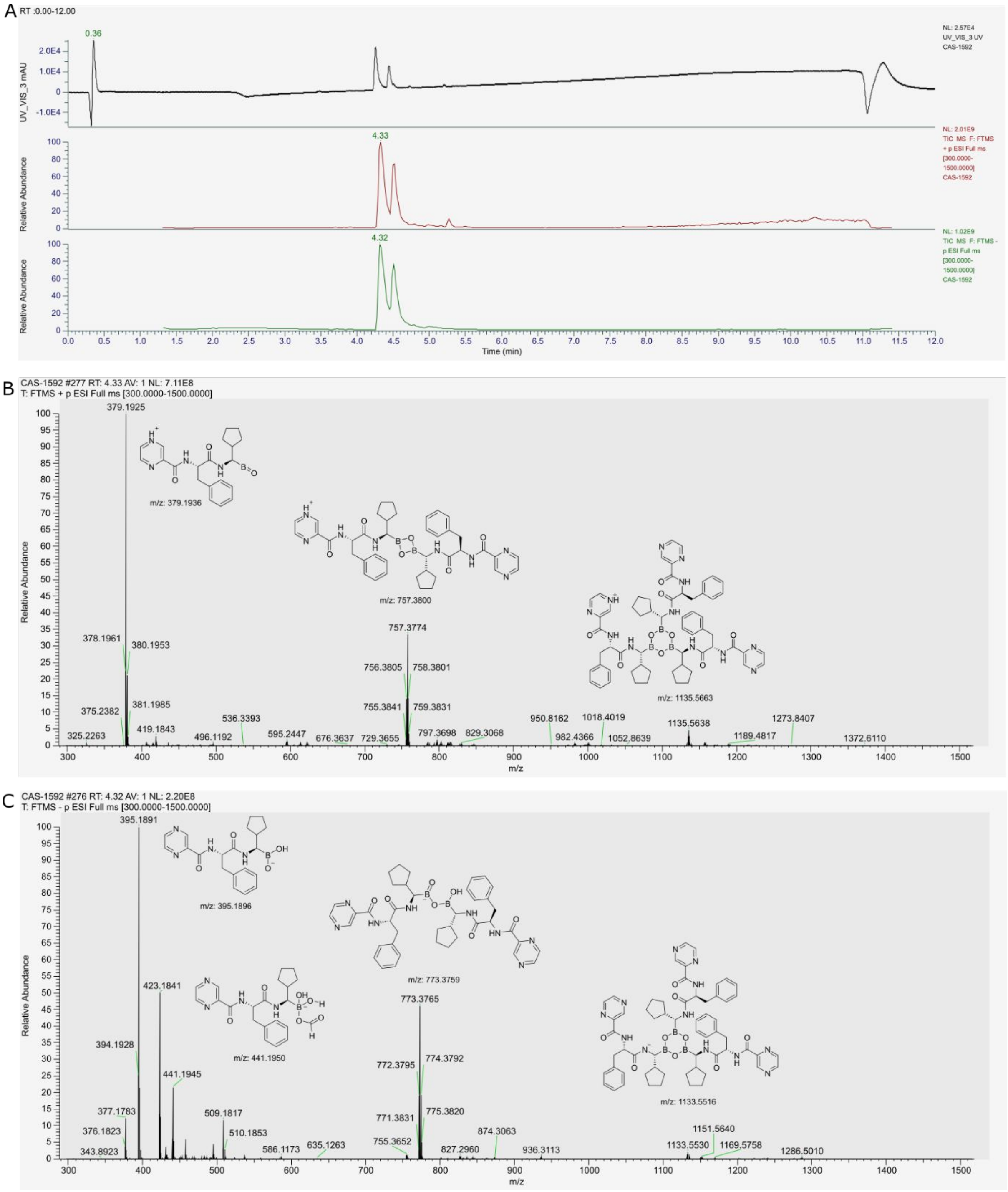

Supporting Figure 2. (A) From top to bottom: chromatogram of compound **5l** recorded at 255 nm, total ion chromatogram (TIC) recorded in positive mode and TIC recorded in negative mode. (B) Positive-ion mass spectrum recorded at 4.33 min. (C) Negative-ion mass spectrum recorded at 4.32 min. Both mass spectra are annotated with the proposed structures of ions derived from compound **5l**, showing calculated monoisotopic m/z values for the most abundant isotopologues.

((*R*)-2-phenyl-1-((*S*)-3-phenyl-2-(pyrazine-2-carboxamido)propanamido)ethyl)boronic acid (5i)

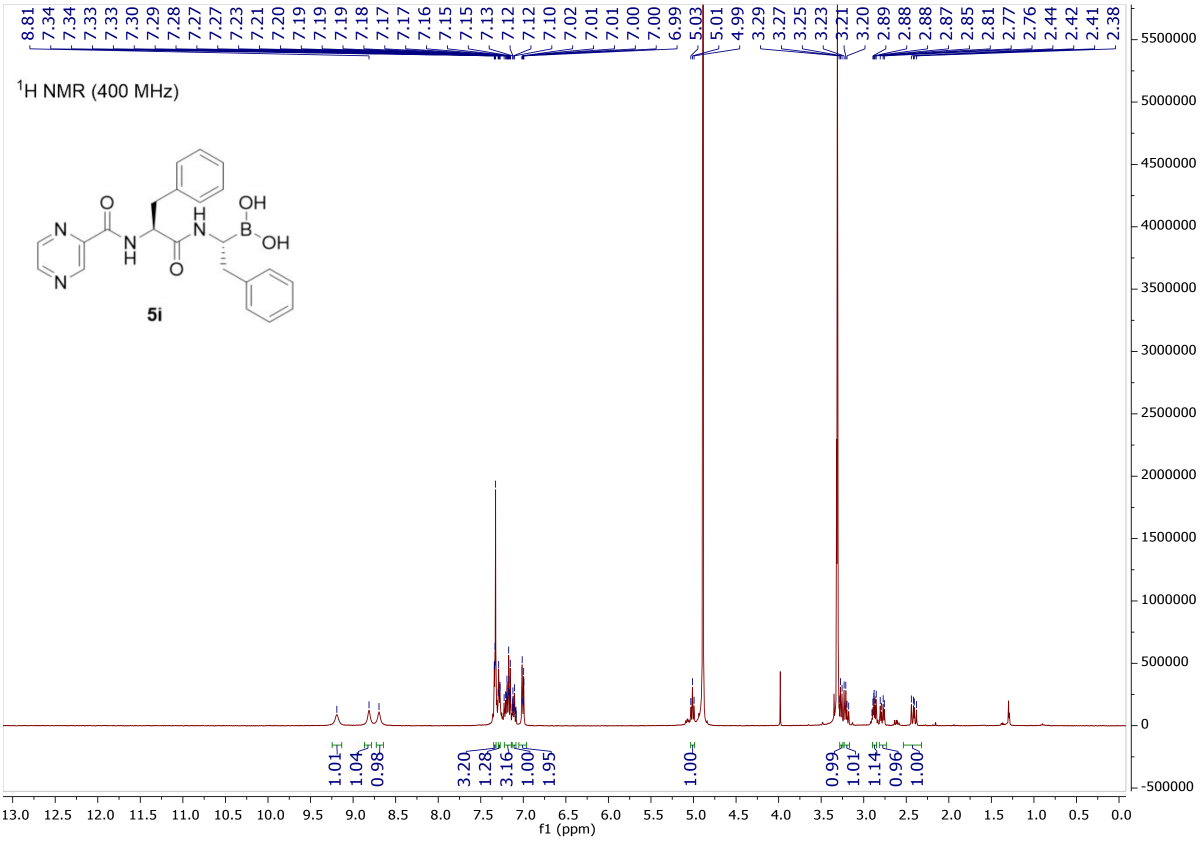

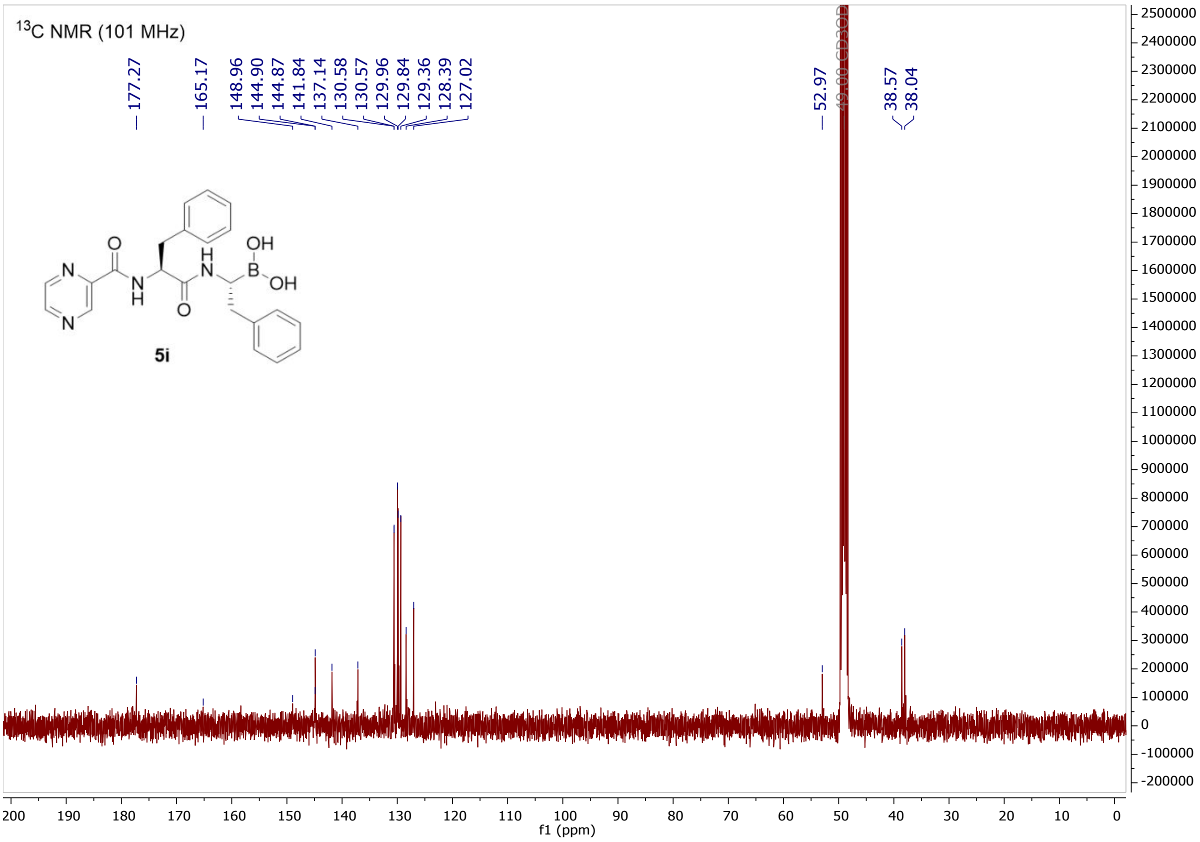

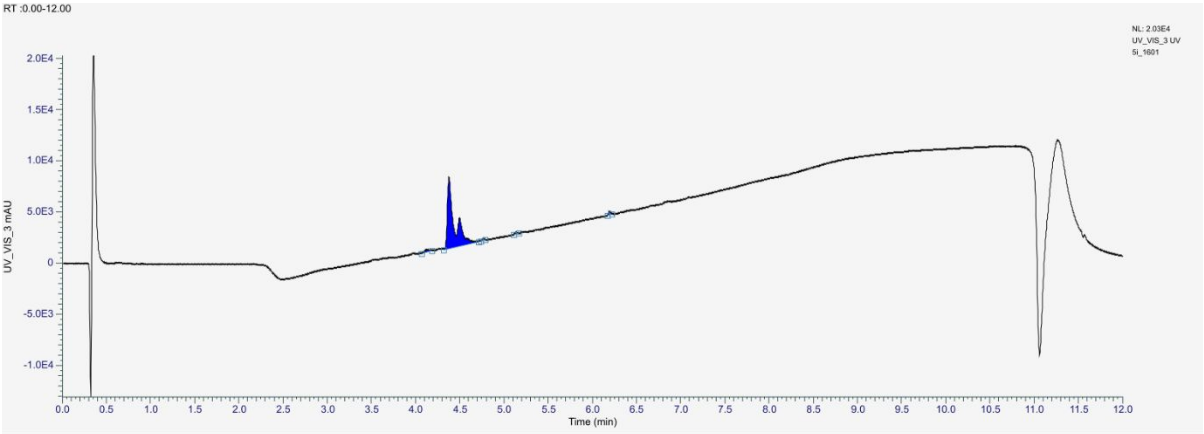

Index	RT (Min)	Peak Area	% Area
1	4.12333333333333	744.254927151551	1.85
2	4.37666666666667	38451.1712077396	95.6
3	4.76333333333333	218.522004782818	0.54
4	5.13666666666667	286.416854121643	0.71
5	6.19666666666667	522.066757229815	1.3

Chromatogram of compound **5i** recorded at 255 nm with integrated peaks.

((*R*)-1-((*S*)-3-phenyl-2-(pyrazine-2-carboxamido)propanamido)propyl)boronic acid (5j)

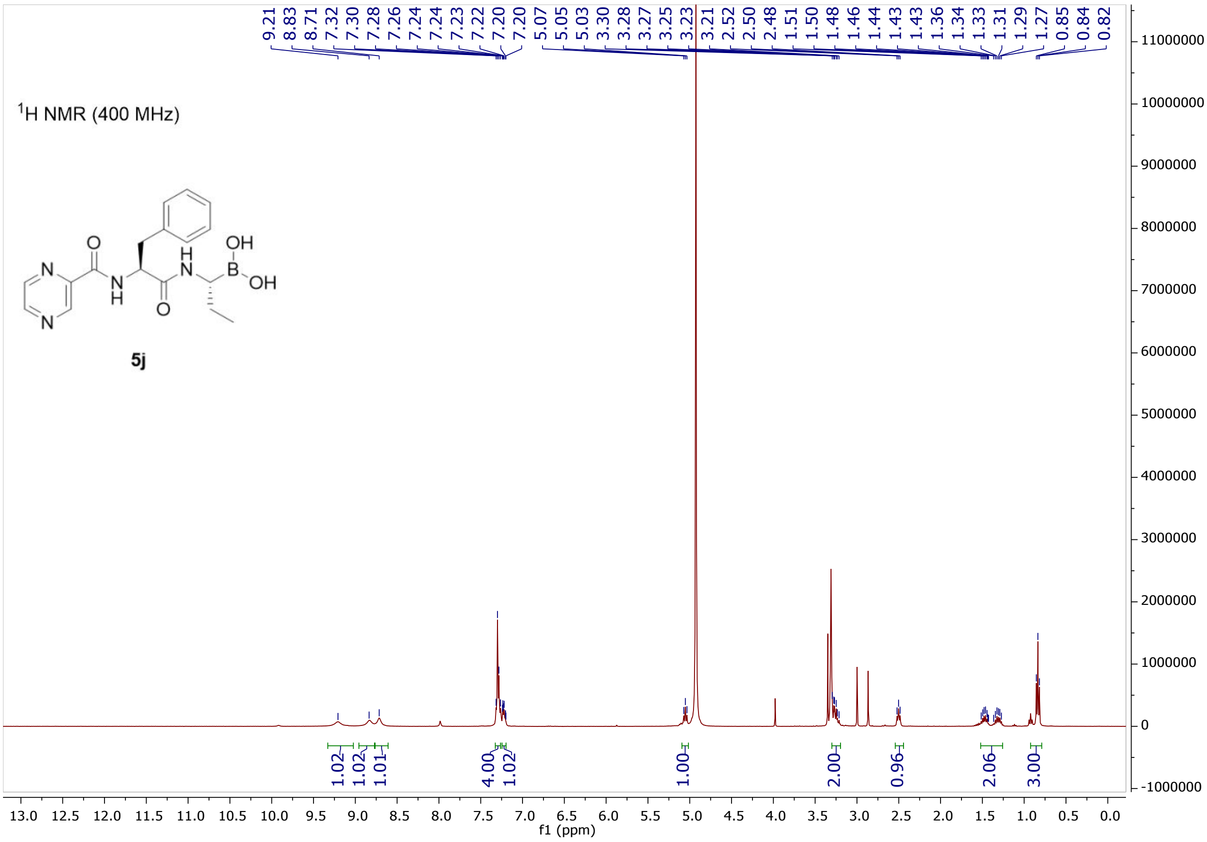

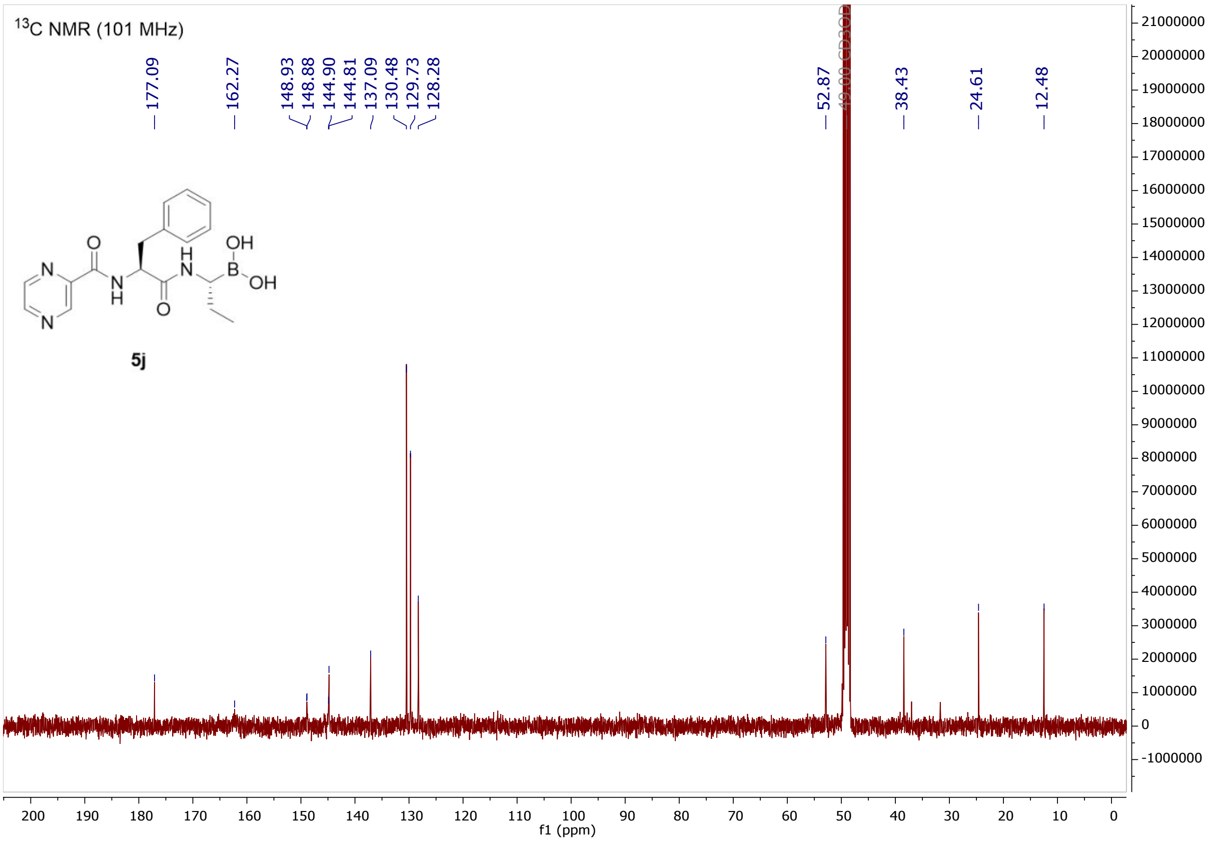

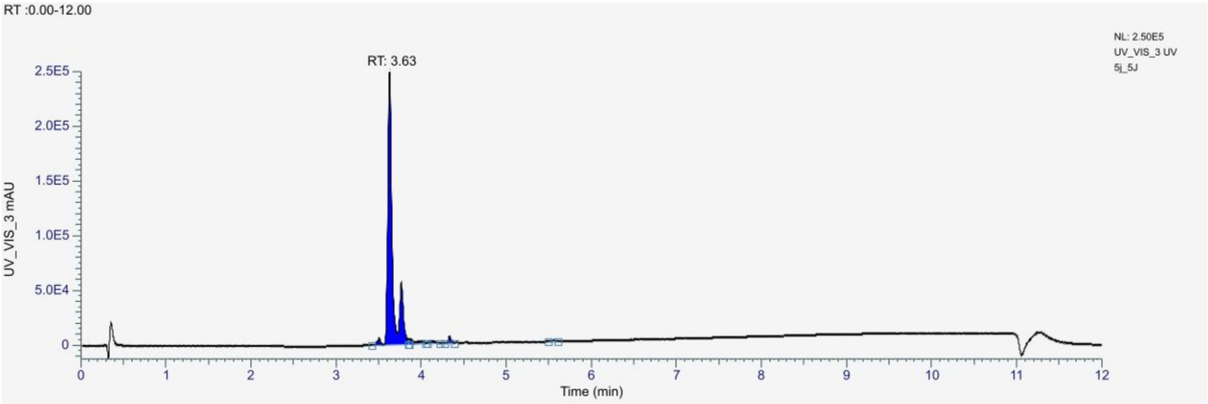

Index	RT (Min)	Peak Area	% Area
1	3.63333333333333	987847.333988198	95.5
2	3.87333333333333	23038.5455890641	2.23
3	4.13333333333333	7641.27161640909	0.74
4	4.33666666666667	13195.324569	1.28
5	5.56	2633.10793	0.25

Chromatogram of compound **5j** recorded at 255 nm with integrated peaks.

((*R*)-cyclopropyl((*S*)-3-phenyl-2-(pyrazine-2-carboxamido)propanamido)methyl)boronic acid (5k)

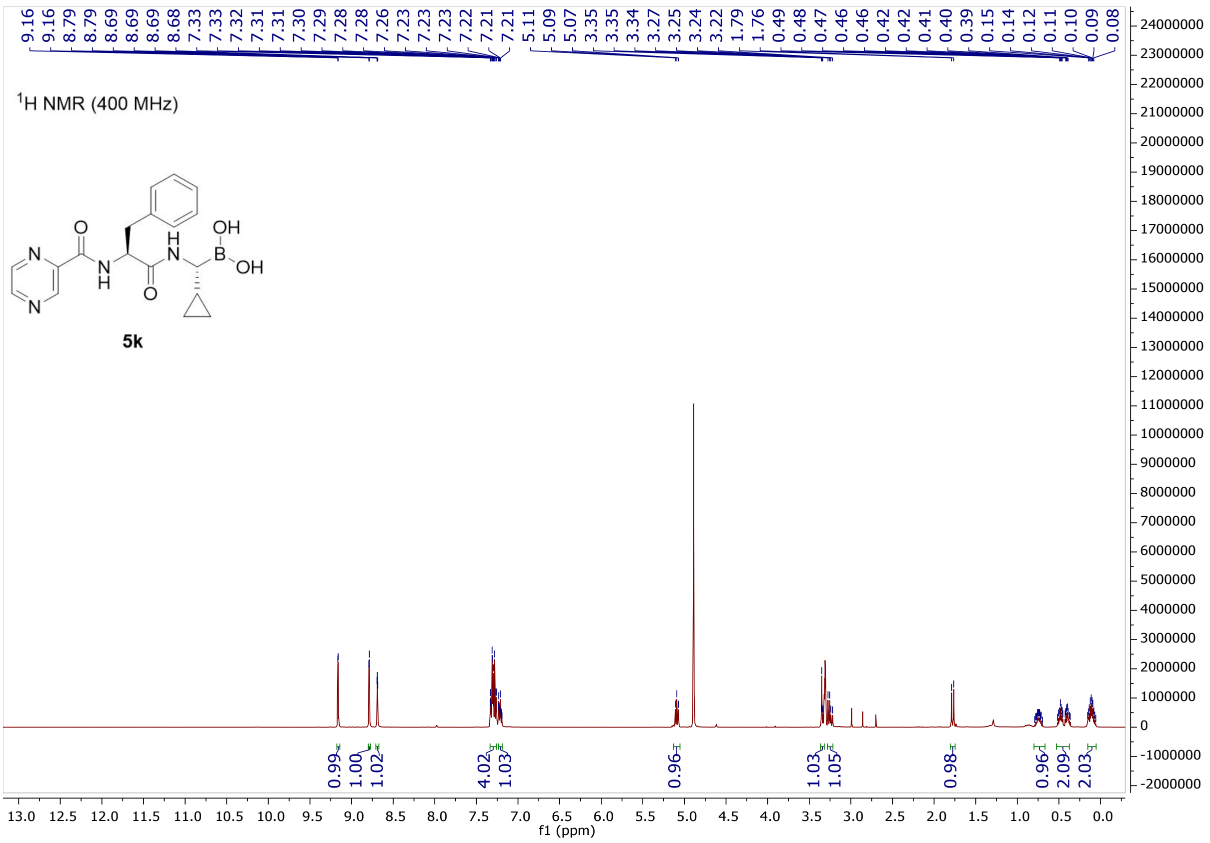

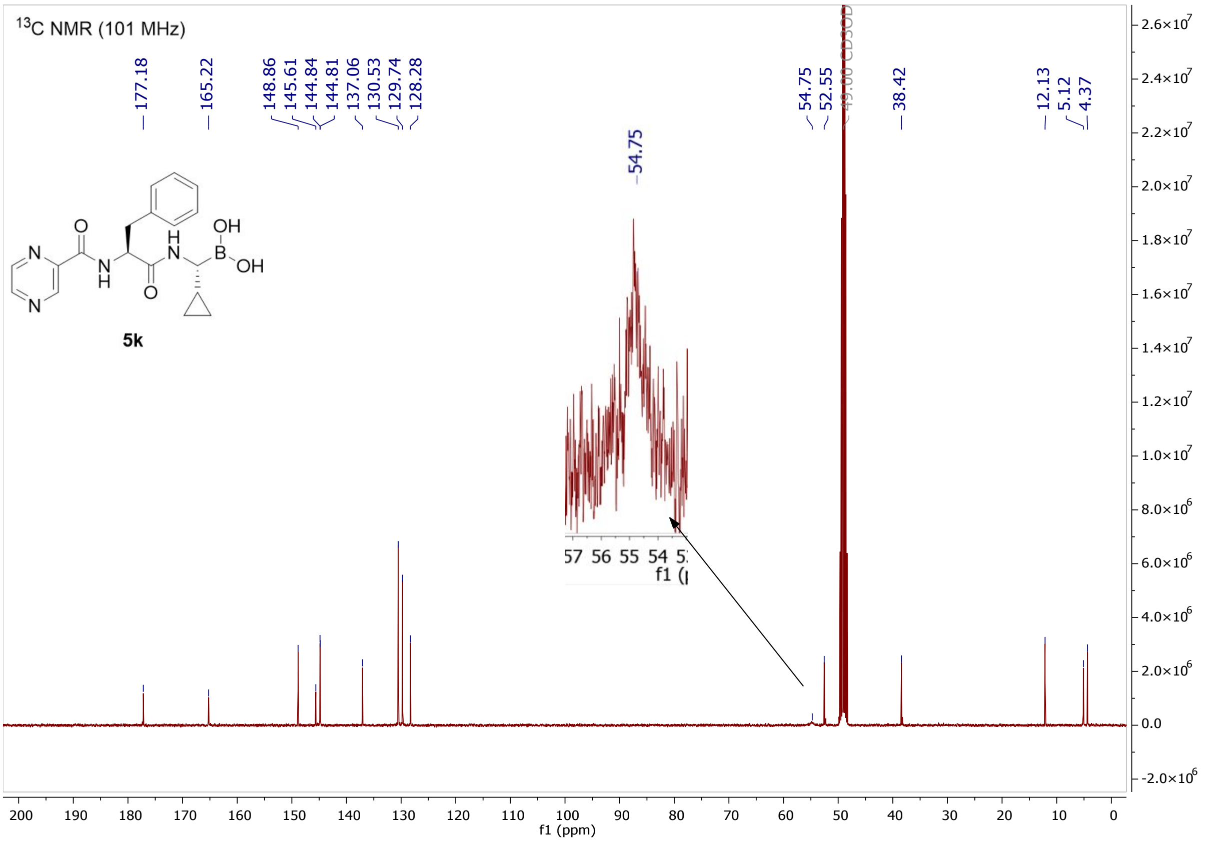

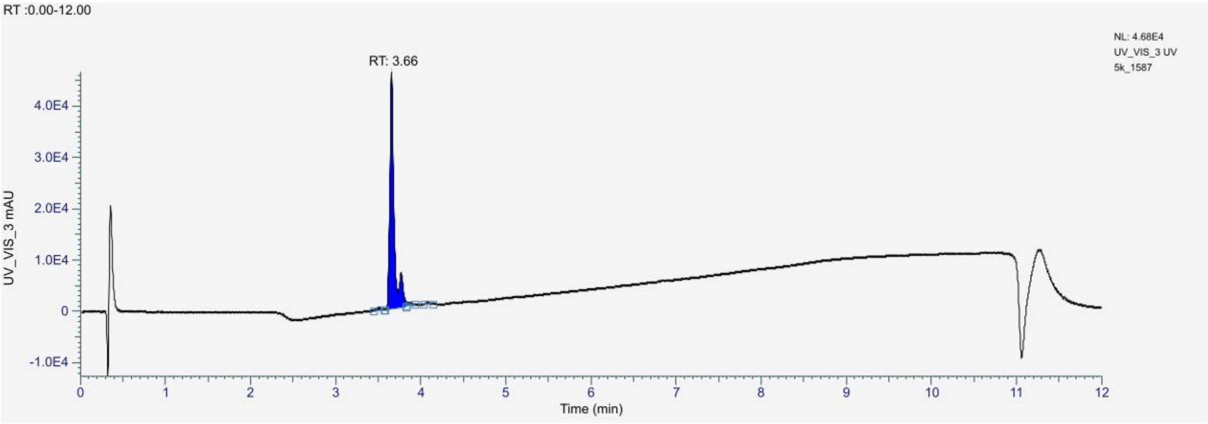

Index	RT (Min)	Peak Area	% Area
1	3.51	1509.91289049128	0.89
2	3.66	166822.649975897	98.14
3	3.83333333333333	1266.49831176316	0.75
4	4.08	382.800090732033	0.23

Chromatogram of compound **5k** recorded at 255 nm with integrated peaks.

((*R*)-cyclopentyl((*S*)-3-phenyl-2-(pyrazine-2-carboxamido)propanamido)methyl)boronic acid (5l)

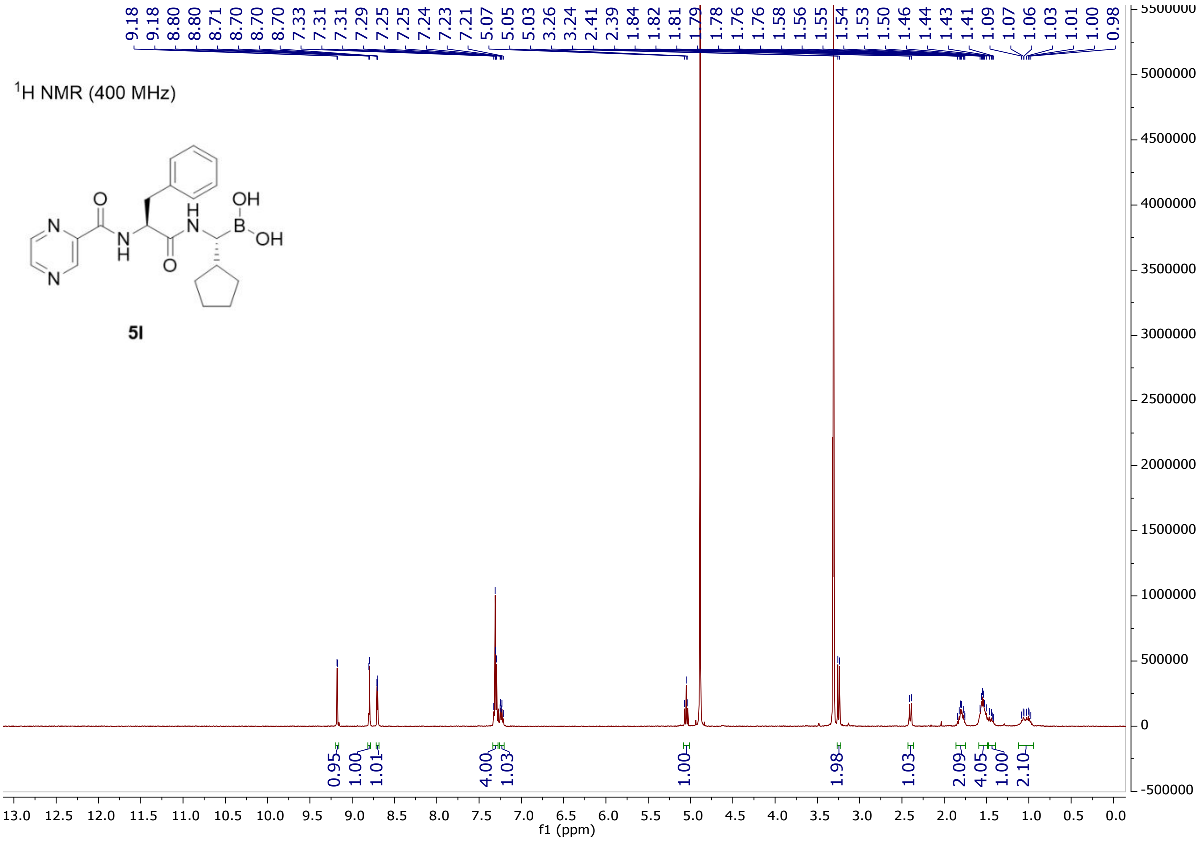

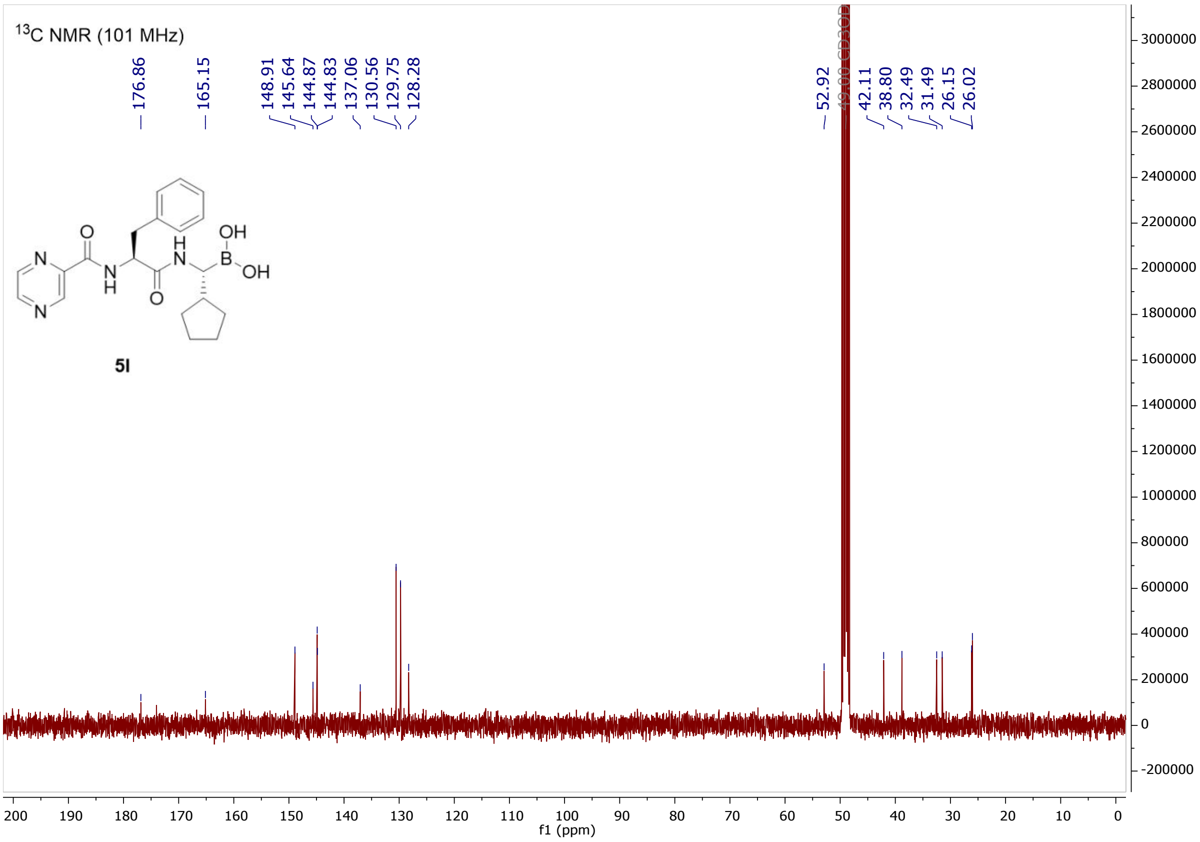

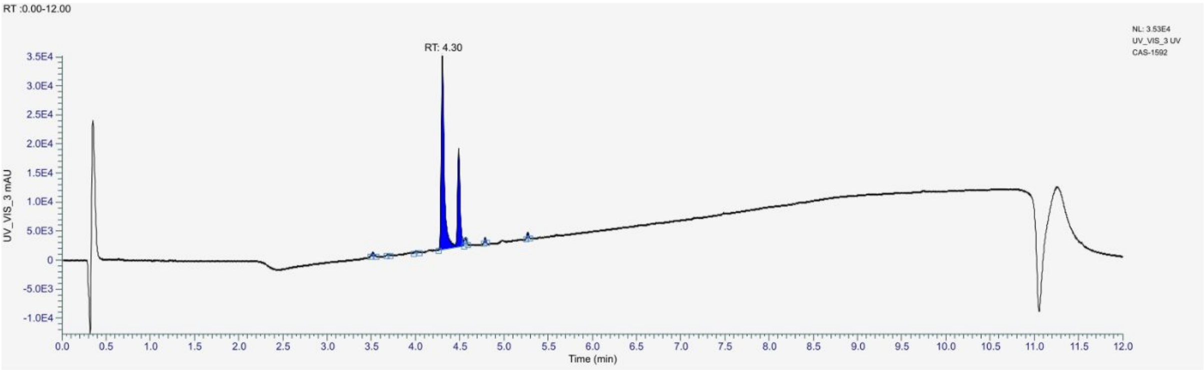

Index	RT (Min)	Peak Area	% Area
1	3.52	1308.57018167543	1.09
2	3.68666666666667	271.740542662267	0.23
3	4.00333333333333	552.865256599997	0.46
4	4.30333333333333	114702.57312047	95.7
5	4.57	709.15846287612	0.59
6	4.78666666666667	1009.44116518311	0.84
7	5.27	1303.93772332804	1.09

Chromatogram of compound **5l** recorded at 255 nm with integrated peaks.

5. References

(1) Huber, E. M.; Groll, M. A Nut for Every Bolt: Subunit-Selective Inhibitors of the Immunoproteasome and Their Therapeutic Potential. *Cells* **2021**, *10*, 1929.

(2) Klein, M.; Busch, M.; Friese-Hamim, M.; Crosignani, S.; Fuchss, T.; Musil, D.; Rohdich, F.; Sanderson, M. P.; Seenisamy, J.; Walter-Bausch, G.; Zanelli, U.; Hewitt, P.; Esdar, C.; Schadt, O. Structure-Based Optimization and Discovery of M3258, a Specific Inhibitor of the Immunoproteasome Subunit LMP7 (β5i). *J. Med. Chem.* **2021**, *64*, 10230–10245.

(3) Bross, P. F.; Kane, R.; Farrell, A. T.; Abraham, S.; Benson, K.; Brower, M. E.; Bradley, S.; Gobburu, J. V.; Goheer, A.; Lee, S.-L.; Leighton, J.; Liang, C. Y.; Lostritto, R. T.; McGuinn, W. D.; Morse, D. E.; Rahman, A.; Rosario, L. A.; Verbois, S. L.; Williams, G.; Wang, Y.-C.; Pazdur, R. Approval Summary for Bortezomib for Injection in the Treatment of Multiple Myeloma. *Clin. Cancer Res.* **2004**, *10*, 3954–3964.

(4) Bettinger, H. F. Reversible Formation of Organyl(Oxo)Boranes (RBO) (R = C6H5 or CH3) from Boroxins ((RBO)3): A Matrix Isolation Study. *Organometallics* **2007**, *26*, 6263–6267.

(5) Parker, D. S. N.; Dangi, B. B.; Balucani, N.; Stranges, D.; Mebel, A. M.; Kaiser, R. I. Gas-Phase Synthesis of Phenyl Oxoborane (C_6H_5BO) via the Reaction of Boron Monoxide with Benzene. *J. Org. Chem.* **2013**, *78*, 11896–11900

(6) Wang, L.; Dai, C.; Burroughs, S. K.; Wang, S. L.; Wang, B. Arylboronic Acid Chemistry under Electrospray Conditions. *Chem. Eur. J.* **2013**, *19*, 7587–7594.
